# White matter alterations in focal to bilateral tonic-clonic seizures

**DOI:** 10.3389/fneur.2022.972590

**Published:** 2022-09-14

**Authors:** Christina Maher, Arkiev D'Souza, Rui Zeng, Michael Barnett, Omid Kavehei, Armin Nikpour, Chenyu Wang

**Affiliations:** ^1^School of Biomedical Engineering, Faculty of Engineering, The University of Sydney, Sydney, NSW, Australia; ^2^Brain and Mind Centre, The University of Sydney, Sydney, NSW, Australia; ^3^Australian Research Council Training Centre for Innovative BioEngineering, The University of Sydney, Sydney, NSW, Australia; ^4^Translational Research Collective, Faculty of Medicine and Health, The University of Sydney, Sydney, NSW, Australia; ^5^Department of Neurology, Royal Prince Alfred Hospital, Camperdown, NSW, Australia; ^6^Sydney Neuroimaging Analysis Centre, Camperdown, NSW, Australia; ^7^Central Clinical School, Faculty of Medicine and Health, The University of Sydney, Sydney, NSW, Australia

**Keywords:** focal to bilateral tonic-clonic seizure, diffusion MRI, track-weighted imaging, tensor-based imaging, focal epilepsy, white matter, microstructural

## Abstract

We examined the white matter of patients with and without focal to bilateral tonic-clonic seizures (FBTCS), and control participants. A neural network based tract segmentation model (Tractseg) was used to isolate tract-specific, track-weighted tensor-based measurements from the tracts of interest. We compared the group differences in the track-weighted tensor-based measurements derived from whole and hemispheric tracts. We identified several regions that displayed significantly altered white matter in patients with focal epilepsy compared to controls. Furthermore, patients without FBTCS showed significantly increased white matter disruption in the inferior fronto-occipital fascicle and the striato-occipital tract. In contrast, the track-weighted tensor-based measurements from the FBTCS cohort exhibited a stronger resemblance to the healthy controls (compared to the non-FBTCS group). Our findings revealed marked alterations in a range of subcortical tracts considered critical in the genesis of seizures in focal epilepsy. Our novel application of tract-specific, track-weighted tensor-based measurements to a new clinical dataset aided the elucidation of specific tracts that may act as a predictive biomarker to distinguish patients likely to develop FBTCS.

## 1. Introduction

Focal to bilateral tonic-clonic seizures (FBTCS) are a feature of focal epilepsy that can lead to an increased risk of cardiac arrhythmias (1), seizure-related injuries (2), and sudden unexpected death in epilepsy (SUDEP) (3). Unlike other focal epilepsies, the mechanisms underlying FBCTS, in particular, remain elusive. Therefore, the importance of delineating the mechanisms involved in FBTCS is amplified as a vital objective to aid the control of FBTCS and the prevention of SUDEP.

Although the taxonomy of FBTCS implies whole-brain generalization of seizures, FBTCS are primarily highly selective, producing more vigorous activity in specific brain regions (4–6). Mounting evidence has endorsed subcortical structures such as the thalamus and basal ganglia and their associated circuits as critical to the information relay involved in FBTCS. The topographical arrangement of the thalamocortical relay fibres allow the projection to the cerebral cortex, from which sensory information is processed and relayed back to the original projection site in the thalamus. Acting as a “relay station” (7), the thalamus has widespread connections across the entire cerebral cortex (8) and moderates communication between various brain regions. Within the context of FBTCS, the thalamus has been proposed as a support system for seizure propagation *via* its role in the synchronization of abnormal cortical-subcortical ictal discharge (9, 10).

Additionally, the basal ganglia functions as a “braking system”, interacting with the thalamus and cortex through multiple parallel circuits, including the direct and indirect pathways (11). The basal ganglia are increasingly hypothesized to play an anticonvulsive role in FBTCS (12), yet the specific mechanisms remain unclear. Increased basal ganglia activity was reported to be negatively associated with FBTCS in TLE (13, 14). In contrast, others illustrated that the basal ganglia only become involved when ictal activity disperses to additional cortical regions during secondary generalization (15).

Tensor-based metrics derived from diffusion tensor imaging have been established as a valuable technique to quantify white matter changes in a range of epilepsies (16, 17). A meta-analysis showed patients with focal epilepsy had elevated regional mean diffusivity relative to controls (17). However, findings of altered fractional anisotropy (FA) are less consistent; some studies report reduced FA in patients with epilepsy compared to controls (18, 19), whilst others report no change across several regions (20, 21); thus, further clarification is required.

Moreover, the majority of diffusion MRI studies focus on TLE without the FBTCS subtype (17). One study that examined structural network alterations in patients with TLE alone vs. TLE with FBTCS used a “node abnormality” metric to quantify patient specific node abnormality load (22). They showed that patients with FBTCS in TLE had increased abnormality load in subcortical regions. However, their network approach did not allow the identification of specific tracts that may be involved in FBTCS. Though FBTCS can present under the umbrella of TLE, a reproducible and reliable method to identify specific tracts that may be involved in FBTCS would yield great clinical utility.

Advances in image analysis techniques, such as tract-weighted tensor-metrics, may improve the reliability of findings and enhance the interpretation of standard tensor-based metrics. A recent advancement to traditional tensor-based measurements is the introduction of track-weighted (TW) measurements. Here, tensor-based images are weighted by reconstructed streamlines traversing a given voxel to generate a TW version of the tensor-based image (23). TW measurements have been shown to improve reproducibility (i.e., TW-FA has significantly higher reproducibility than FA) and, for a given effect size, have increased power to detect group differences (23). Therefore, TW tensor-based measurements (termed “TW-TM” for brevity) could reveal subtle differences in epilepsy subtypes that would typically be undetected if quantified using only traditional tensor-based metrics.

Since the subcortical structures involved in FBTCS are also involved in focal seizures (24), we included both subtypes (focal only and focal with FBTCS) in this study to uncover distinct structural differences between the two groups. In addition to the thalamic and striatal regions, we included other white matter regions based on their well-documented role in focal seizures and functional connectivity (18, 25), to determine whether any observed group differences were unique to the FBTCS group, and to account for possible whole brain differences.

The innovation in this study is the combination of a neural network based tract segmentation model [Tractseg; (26)], with subject specific, TW-TM, to investigate specific tracts as potential biomarkers of seizure propagation in FBTCS. We hypothesized that compared to controls, patients with focal epilepsy would have significantly altered white matter in the subcortical regions, as observed through the TW-TM. We further hypothesized that compared to those without FBTCS, patients with FBTCS would have significantly altered white matter in the thalamic and striatal regions, as observed through the TW-TM.

## 2. Methods

### 2.1. Participants and data

Twenty-seven adults with focal epilepsy were recruited from the Comprehensive Epilepsy Centre at the Royal Prince Alfred Hospital (RPAH, Sydney, Australia); and MRI was performed at the Brain and Mind Centre (Sydney, Australia). Inclusion criteria were adults diagnosed with focal epilepsy, aged 18–60, presenting without surgery, and with or without a cortical brain lesion, who were willing and able to comply with the study procedures for the duration of their participation. Exclusion criteria were pregnant women and individuals with intellectual disabilities. The 20 controls were neurologically normal individuals. Written informed consent was obtained from all participants before study participation. Ethical approval was obtained from the RPAH Sydney Local Health District (RPAH-SLHD) ethics committee (RPAH-SLHD approval ID: X14-0347, HREC/14/RPAH/467). All research and methods were performed in accordance with the Declaration of Helsinki and conducted in accordance with the relevant guidelines and regulations prescribed by the RPAH-SLHD.

#### 2.1.1. Participant groups

The participants were placed into the following groups:

1. “All patients”: The entire cohort of 27 patients; all diagnosed with focal epilepsy. The “All patients” group was further separated into the following two subgroups:
  - “FBTCS-Y”: In this subgroup, the 19 patients were defined as having frequent (more than two per year) or infrequent (one per year) large, homolateral and simultaneous FBTCS. The FBTCS may have occurred during observation at the RPAH Epilepsy Centre or reported by the participant as occurring elsewhere.
  - “FBTCS-N”: In this subgroup, the 8 patients had never experienced FBTCS.
2. “Controls”: The control group consisted of 20 age and sex matched healthy individuals with no history of epilepsy.

### 2.2. Image acquisition

All scans were acquired on the same GE Discovery^TM^ MR750 3T scanner (GE Medical Systems, Milwaukee, WI). For each participant, the following sequences were acquired: Pre-contrast 3D high-resolution T1-weighted image (0.7 mm isotropic) using fast spoiled gradient echo (SPGR) with magnetization-prepared inversion recovery pulse (TE/TI/TR = 2.8/450/7.1 ms, flip angle = 12); and axial diffusion-weighted imaging (2 mm isotropic, TE/TR = 85/8,325 ms) with a uniform gradient loading (*b* = 1,000 s/mm^2^) in 64 directions and 2 *b*0 s. An additional *b*0 image with reversed phase-encoding was also acquired for distortion correction (27).

### 2.3. Image preprocessing

The T1 images were processed using a modified version of Freesurfer's recon-all (v6.0) (28), alongside an in-house skull-stripping tool (Sydney Neuroimaging Analysis Centre). Each subject was inspected, and minor segmentation errors were manually corrected. A 5 tissue-type (5TT) image (29) was generated using MRtrix3 (30). The T1 image was registered to the mean *b*0 image; the warp was used to register the 5TT image to the diffusion image.

Diffusion image processing was conducted using MRtrix3 (30). The diffusion pre-processing included motion and distortion correction (27, 31), bias correction using ANTs (32), and resizing to voxel size 1 mm isotropic. The *dhollander* algorithm (33) was used to estimate the response functions of the white matter, gray matter, and cerebral spinal fluid, from which constrained spherical deconvolution was used to estimate the fibre orientation distributions using MRtrix3Tissue (30). The intensity of the white matter fibre orientation distributions was normalized (30), and used for anatomically constrained whole-brain tractography (34) (along with the registered 5TT image). The tractography protocols were as follows: 15 million tracks were generated, iFOD2 probabilistic fibre tracking (35), dynamic seeding (36), maximum length 300 mm, backtrack selected and crop at gray-matter-white-matter interface selected. For quantitative analysis, the corresponding weight for each streamline in the tractogram was derived using SIFT2 (36); the streamline weights and tractogram were used to generate a track-density image (TDI) (37).

#### 2.3.1. Track-weighted tensor-based measurements

The pre-processed diffusion image was used to calculate the diffusion tensor. By resolving the tensor into its primary, secondary, and tertiary directions of diffusion, the following tensor-based metrics were calculated: apparent diffusion coefficient (ADC, the average of the three directions of diffusion), fractional anisotropy (FA, degree of anisotropy ranging from 0 to 1, whereby 0 indicates isotropic diffusion and 1 indicates diffusion exclusively along a single axis), axial diffusivity (AD, the primary direction of diffusion, representative of diffusion parallel to axonal fibres) and radial diffusivity (RD, the average of the secondary and tertiary directions of diffusion, representing diffusion perpendicular to axonal fibres) (38). Next, the tracks (and their weights) were used to calculate the TW-TM (i.e., TW-ADC, TW-FA, TW-AD, and TW-RD) (39). The following settings were used to generate TW images: Gaussian statistic, full-width-half-maximum of 40 and voxel size of 0.2mm.

#### 2.3.2. Measuring tract-specific, track-weighted tensor-based metrics

**Figure 1:**
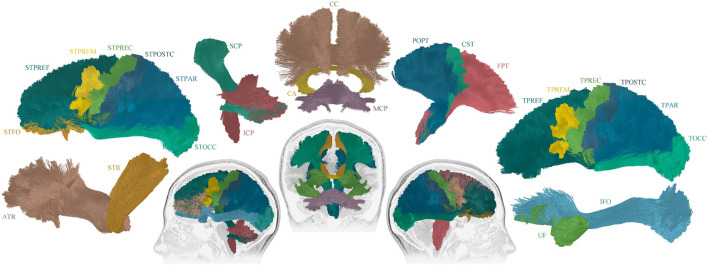
Overview of the 25 reconstructed tracts of interest. The following tracts were included (tracts names were assigned following (26): Thalamo - Prefrontal (TPREF), Premotor (TPREM), Precentral (TPREC), Postcentral (TPOSTC), Parietal (TPAR), and Occipital (TOCC); Striato - Fronto-Orbital (STFO), Prefrontal (STPREF), Premotor (STPREM), Precentral (STPREC), Postcentral (STPOSTC), Parietal (STPAR), and Occipital (STOCC); Anterior thalamic radiation (ATR), Superior thalamic radiation (STR), Corticospinal (CST), Fronto pontine (FPT), Parieto-occipital pontine (POPT), Inferior cerebellar peduncle (ICP), Middle cerebellar peduncle (MCP), Superior cerebellar peduncle (SCP), Inferior fronto-occipital fascicle (IFO), Uncinate fascicle (UF), Commissure anterior (CA), and Corpus callosum (CC). For tracts that exist in both hemispheres, the right and left sides were included in the image construction, though only the sagittal view is displayed. The tracts that cross between the hemispheres are displayed in coronal view.

**Figure 2:**
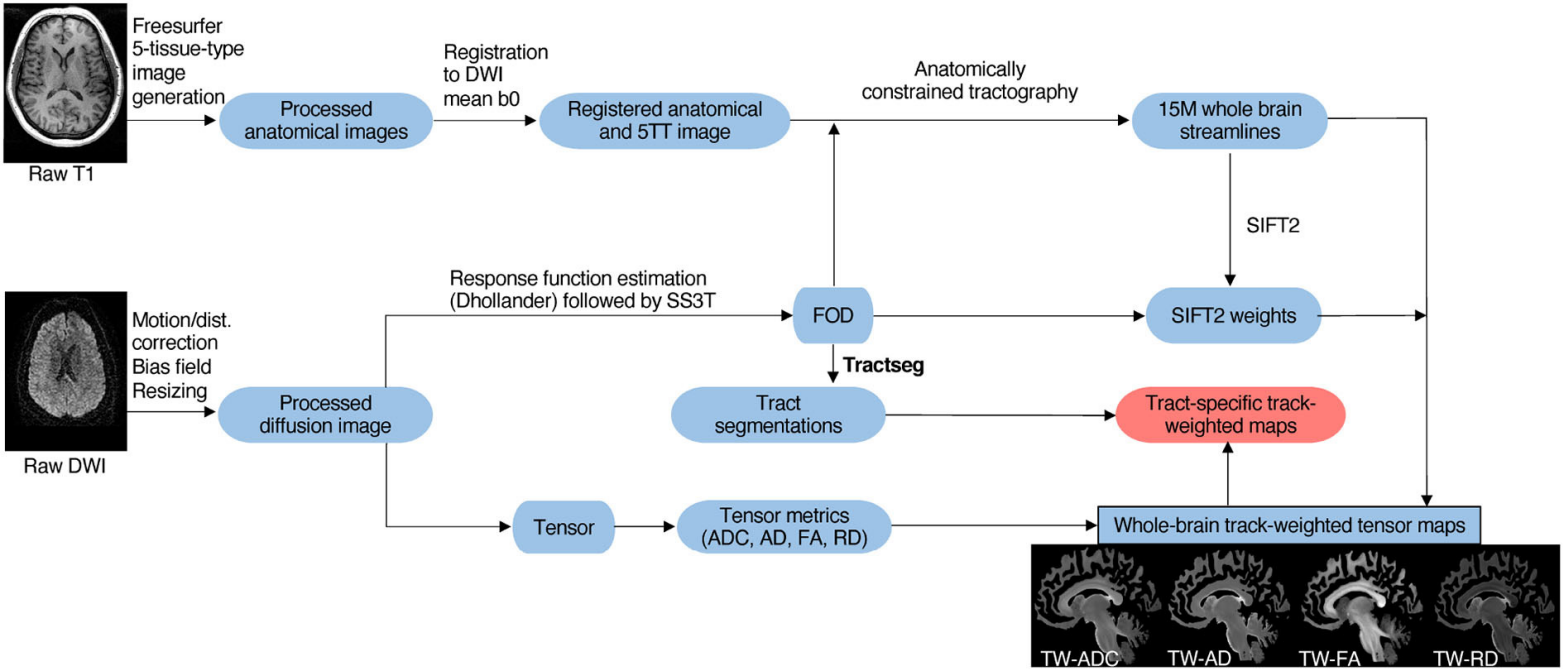
Schematic overview of imaging analysis pipeline. As described in Section 2.3, T1 and diffusion MRIs were preprocessed. Next, tract generation and subject-specific tract segmentation was conducted. Lastly, tensor maps were produced, and the tract-specific, track-weighted tensor-based measurements were derived.

Tractseg (26), a convolutional neural network based, automated tract segmentation model, was used to obtain data-driven, subject-specific tract segmentations. The tracts were used to isolate TW-TM in the specific tracts of interest. The tracts of interest are shown in Figure 1, and a schematic flow chart of the image analysis pipeline is in Figure 2.

#### 2.3.3. Quality control

A neuroimaging specialist (CW) and neuroimaging researcher (CM) conducted quality control checks by visually inspecting each tract for consistency and anatomical correctness. The cerebellum areas in each subject were inspected and registration accuracy was verified. The right TPAR tract failed the quality control check in 10 participants; therefore the whole tract was removed from the analysis. Twenty-four tracts were included in the final statistical analysis.

### 2.4. Statistical analysis

#### 2.4.1. Demographics

The chi-square test was used to investigate the existence of the following relationships in the patient subgroups: lesion presence and seizure onset side; lesion presence and FBTCS. Chi-square also tested for differences in drug resistance between patient subgroups and gender differences between the patient subgroups and controls. Analysis of variance (ANOVA) tests were conducted to examine differences in age between the patient subgroups and controls, and disease duration between the patient subgroups.

#### 2.4.2. Group comparisons: All patients vs. controls

To examine whole-tract differences between groups, the weighted average of the left and right side of each tract was calculated using the equation [WeightedAverage-TW-TM = ((*L*_TW−TM_ × *L*_count_) + (*R*_TW−TM_ × *R*_count_))/(*L*_count_ + *R*_count_)] (TW-TM: track-weighted tensor-based measurement, i.e., TW-ADC; count: tract count). Univariate analysis of covariance (ANCOVA) tests, which are robust to multiple comparisons including covariates (40), were used to examine the whole-tract differences for each TW-TM (i.e.,TW-ADC and TW-FA) between the “All patients” and control groups. A separate set of ANCOVAs was employed to investigate group differences in TW-TM in a given hemisphere. Here, the TW-TM for a given tract from a given hemisphere was compared between the “All patients” and control groups.

#### 2.4.3. Group comparisons: FBTCS-Y, FBTCS-N vs. controls

ANCOVAs were used to investigate differences between the patient subgroups (FBTCS-Y and FBTCS-N) and control participants. Again, the weighted average of each tract's left and right sides was computed (as described above) to investigate the whole-tract differences in TW-TM between the three groups. ANCOVAs were then used to investigate group differences in a given hemisphere. Here, the TW-TM for a given tract from a given hemisphere was compared between the three groups.

#### 2.4.4. Group comparisons: Drug resistant vs. drug responsive patients

It has been shown that drug resistant patients may have widespread alterations (41). To address this possibility in our cohort, the patients were split into “drug resistant” (*N* = 17) and “drug responsive” (*N* = 8) groups. To test for group differences, a multivariate analysis of covariance (MANCOVA) was conducted per TW-TM. Another separate MANCOVA was conducted to test for differences between the FBTCS-Y and FBTCS-N groups, with drug resistance as a covariate.

To account for age effects on diffusivity measures (42), age was included as a covariate in all the ANCOVAs for each above-mentioned group comparison. The threshold for statistical significance was set at *p* = 0.05 for all ANCOVAs. Bonferroni correction was used to correct for multiple comparisons as it has been shown to be a robust statistical method to control for multiple comparisons in the specific context of diffusion tensor imaging and tensor based metrics derived from an epilepsy cohort (25). In each group comparison of the TW-TM, the mean difference, *p*-value, and confidence intervals were taken from the Bonferroni adjusted pairwise comparisons generated from the estimated marginal means, which is robust against unbalanced groups and multiple comparisons. All statistical analysis were conducted in SPSS v28.

## 3. Results

### 3.1. Demographics

Twenty-five patients (10M, 15F, mean age 40 ±12.7 years, 17 with FBTCS-Y and 8 with FBTCS-N) and 19 controls (4M, 15F, mean age 37 ±11.12 years) were included in this study after passing the quality control (QC) check described in the Methods section 2.3. There were no significant differences in age [*F*_(2,41)_ = 1.506, *p* = 0.234] or gender [$X_{\left(2,44\right)}^{2}$ = 1.820, *p* = 0.402] between the FBTCS-Y, FBTCS-N, and control groups. There was no significant difference in drug resistance classification between the FBTCS-Y and FBTCS-N groups, [$X_{\left(1,25\right)}^{2}$ = 1.752, *p* = 0.186]. There was no significant difference in disease duration between the FBTCS-Y and FBTCS-N groups, [*F*_(1,23)_ = 0.121, *p* = 0.731]. There was no significant association between lesion presence and seizure onset side or between lesion presence and FBTCS. Table 1 shows the characteristics of all the participants.

**Table 1:** Participants' demographic and clinical characteristics.

	**FBTCS-Y** **(19)**	**FBTCS-N** **(8)**	**Controls** **(20)**
Age* (M ± SD)	38 ± 10.88	46 ± 15.21	37 ± 11.42
Sex (M / F)	8 / 11	3 / 5	5 / 15
Onset side (L / R / U)	8 / 9 / 2	3 / 3 / 2	NA
Onset age (M ± SD)	18 ± 14.36	30 ± 20.96	NA
Disease duration (M ± SD)	27 ± 16.14	22 ± 12.31	NA
Drug resistant (Y / N)	14 / 5	4 / 4	NA
Auras	11	7	NA
MRI findings			
Normal	11	4	NA
MCD	4	1	NA
CD/FCD	2	1	NA
Hippocampal cyst	1 (R)	1 (L)	NA
Various (PVH, DNET)	1	1	NA
Epilepsy classification			
Frontal (L / R)	2 / 2	0	NA
Temporal (L / R)	1 / 2	0	NA
Parietal (L / R)	1 / 1	2 (R)	NA
Occipital (L / R)	1 / 1	1 (L)	NA
Frontotemporal (L / R / U)	3 / 2	1 / 1 / 1	NA
Frontocentral (L / R)	1 (L)	1 (L)	NA
Unknown	2	1	NA

Key: L, left; R, right, U, unknown; “*”, age at MRI scan; CD, cortical dysplasia. DNET, dysembryoplastic neuroepithelial tumor; FCD, focal cortical dysplasia; MCD, malformations of cortical development; PVH, periventricular heterotopia. Two patients and one control were removed after failing the QC tests.

### 3.2. All patients vs. controls

Individual ANCOVA tests were conducted to examine the between-group differences in the two grouping conditions (“All patients” vs. controls; FBTCS-Y and FBTCS-N vs. controls).

To focus on the results that may yield clinical value, only the TW-TM that displayed a mean significant difference between the groups are reported here and in the following sections (*p*-values extracted from the pairwise comparisons); all other results are reported in the Supplementary Tables. Where more than five tracts showed significant between-group differences for a given TW-TM, the *p*-value is reported as *p* < 0.05. The exact *p*-values and confidence intervals for the ANCOVAs (except for Section 3.4) are provided in the Supplementary Tables.

**Figure 3:**
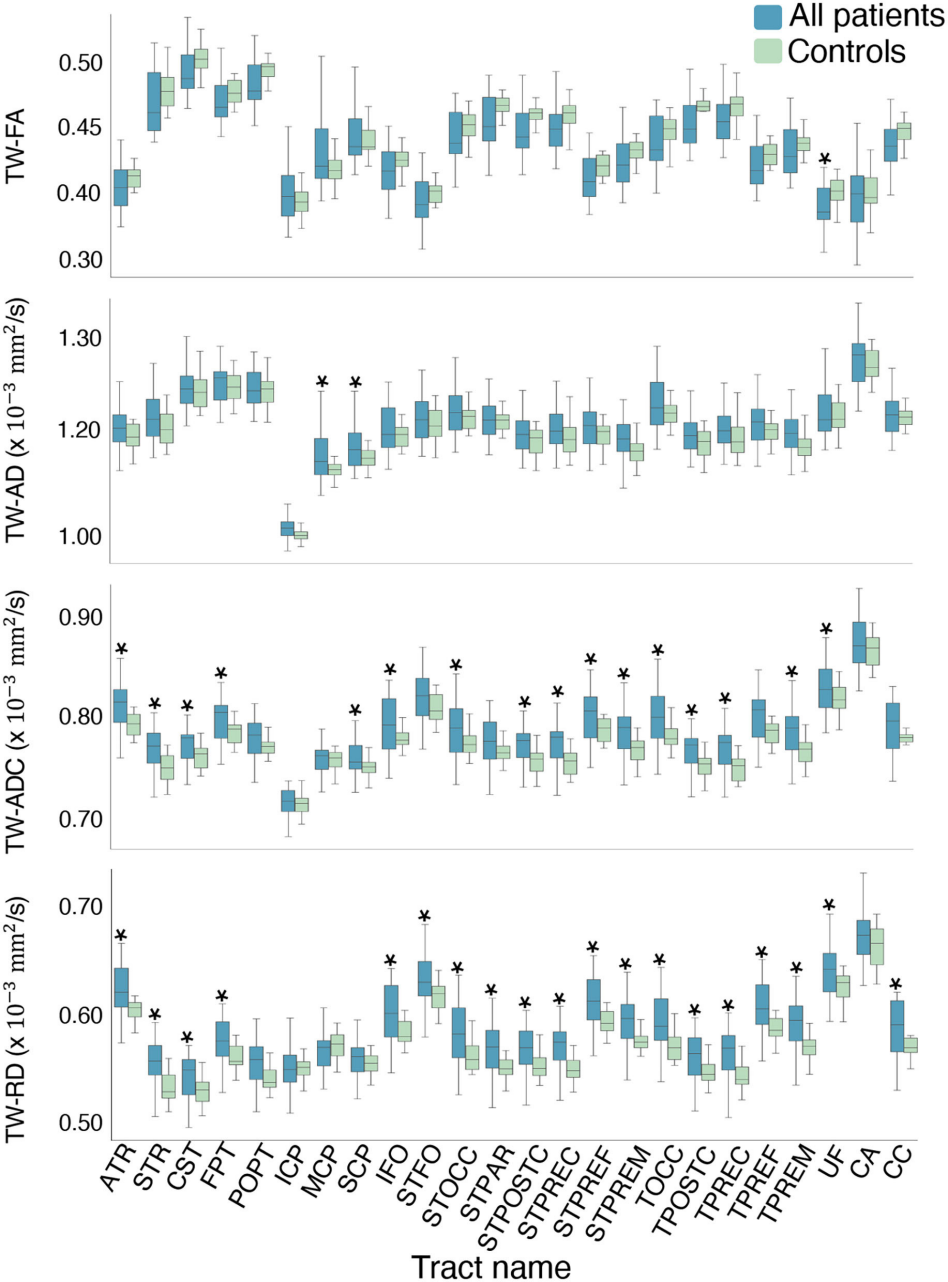
Boxplots of the mean difference in TW-TM for each whole brain tract. The asterisks indicate the tracts where the mean TW-TM difference between the two groups was significant at *p* < 0.05. Notable results that support the hypothesis include the significantly higher mean TW-ADC and TW-RD in the striatal and thalamic tracts (STFO, STOCC, STPAR, STPOSTC, STPREC, STPREF, STPREM, TOCC, TPOSTC, TPREC, TPREF, and TPREM), and the corpus callosum (CC) of the “All patients” group compared to controls. Compared to controls, the “All patients” groups also showed higher TW-ADC and TW-RD in the anterior and superior thalamic radiations (ATR, STR), corticospinal tract (CST), and fronto-pontine (FPT). Compared to controls, the “All patients” groups displayed higher TW-AD in the middle and superior cerebellar peduncles, and lower TW-FA in the uncinate fasciculus (UF).

Compared to controls, the mean TW-ADC was higher in the “All patients” group for 16 out of 24 whole brain tracts (*p* < 0.05). The uncinate fasciculus (UF) had a lower average TW-FA in the “All patients” group compared to controls (*p* = 0.023, partial *n*^2^ = 0.123). Nineteen tracts showed a higher average TW-RD in the “All patients” group compared to controls (*p* < 0.05). The middle and superior cerebellar peduncles (MCP and SCP) had higher mean TW-AD in “All patients” compared to controls (MCP: *p* = 0.033, partial *n*^2^ = 0.108; SCP: *p* = 0.032, partial *n*^2^ = 0.110). Figure 3 contains boxplots of the results. When group differences were tested in each hemisphere, the previously observed significant whole-tract differences were not preserved in both hemispheres. The Bonferroni corrected mean differences, exact *p*-values, effect sizes, and confidence intervals from the ANCOVAs between the “All patients” and control groups are reported in Supplementary Tables 1, 2.

### 3.3. FBTCS-Y, FBTCS-N vs. controls

**Figure 4:**
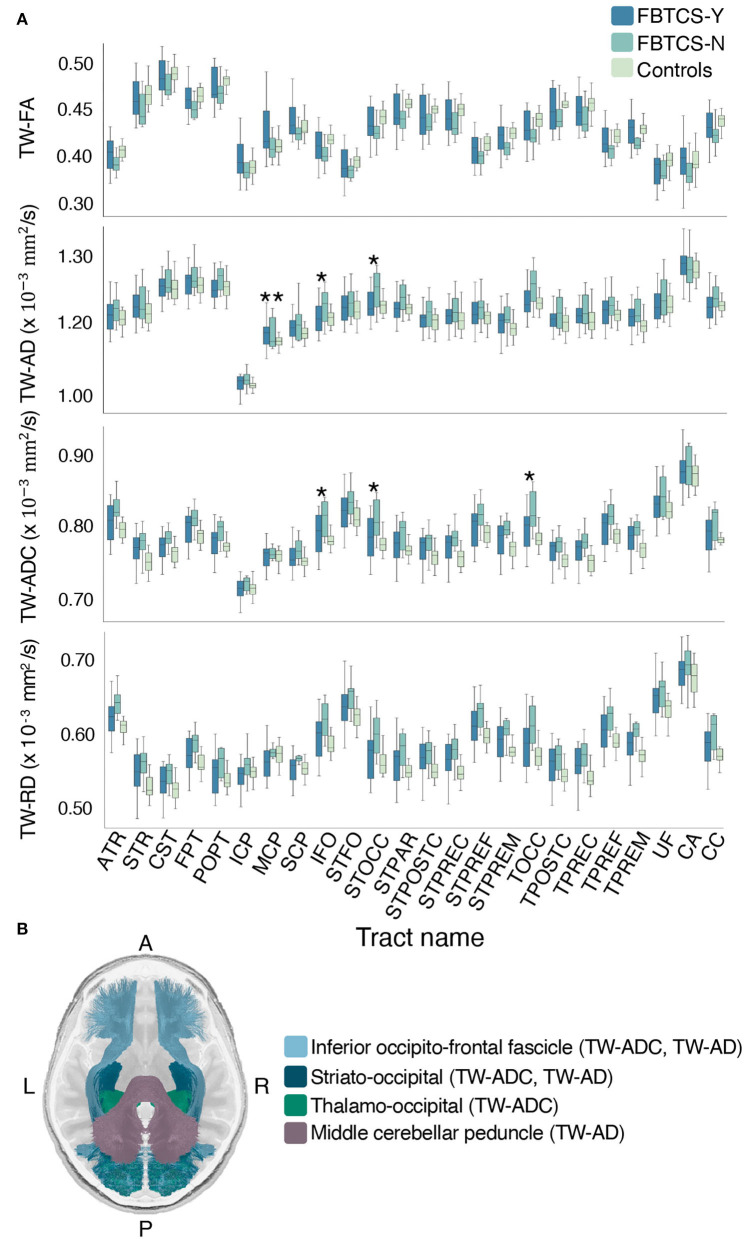
Mean group differences in TW-TM for each whole brain tract. Grouped boxplots for each TW-TM for each whole brain tract are shown in **(A)**. A single asterisk denotes the tracts where the mean TW-TM difference between the two patient groups was significant (*p* < 0.05), whereas two asterisks denote the tracts where the mean TW-TM difference was significant between the FBTCS-Y and control groups (*p* < 0.05). In panel **(B)** is a visual representation of the whole-tracts where a significant difference in mean TW-TM was observed between the two patient groups (light blue: inferior fronto-occipital fascicle, metrics: TW-ADC and TW-AD; dark blue: striato-occipital tract, metric: TW-ADC and TW-AD; green: thalamo-occipital tract, metric: TW-ADC). Also shown in panel **(B)** is the middle cerebellar peduncle (MCP, purple) where a significant difference in mean TW-AD was observed between the FBTCS-Y and control groups. Notably, the mean TW-AD of the middle cerebellar peduncle was significantly different between all patients and controls. Splitting the patients into their subgroups revealed the significant difference was driven by the FBTCS-Y group.

Group differences were observed in a number of TW-TM in the whole-tract comparison. Here, we report the significant *p*-values from the *post-hoc* tests which revealed that overall, the differences between the FBTCS-N and control groups drove the significant effects observed in the ANCOVAs (see Supplementary Table 3). When compared to controls, the FBTCS-N group had higher mean TW-ADC in 18 out of 24 tracts (*p* < 0.05), and in the TW-FA of the corpus callosum (CC) (*p* = 0.043), thalamo prefrontal and premotor tracts (TPREF, TPREM, *p* = 0.048 and *p* = 0.031, respectively). When compared to controls, the FBTCS-N group also had higher mean TW-RD in 18 out of 24 tracts (*p* < 0.05) and higher mean TW-AD in the inferior fronto-occipital fascicle (IFO, *p* = 0.030), and the occipital striato and thalamo tracts (STOCC, TOCC, *p* = 0.018 and *p* = 0.026, respectively). Interestingly, compared to the FBTCS-Y group, the FBTCS-N group showed significantly higher mean TW-TM in the inferior fronto-occipital fascicle, and occipital striato and thalamic tracts (TW-ADC: *p* = 0.044, *p* = 0.032, *p* = 0.042 respectively; TW-AD: IFO: *p* = 0.049, STOCC: *p* = 0.033). The FBTCS-Y group also had significantly higher TW-AD than the controls in the middle cerebellar peduncle (MCP, *p* = 0.042). Figure 4A displays the boxplots of the results, and Figure 4B contains a visual representation of the tracts that exhibited significant TW-TM differences between the patient subgroups, and between the FBTCS-Y group and controls. When group differences were compared hemispherically, the previously observed significant whole-tract differences were not preserved in both hemispheres. The Bonferroni corrected mean differences, exact *p*-values, and confidence intervals from all ANCOVAs between the patient subgroups and control group are reported in Supplementary Tables 3, 4.

Compared to the FBTCS-Y group, the significantly higher TW-ADC and TW-AD of the IFO tract in the FBTCS-N group was retained only in the right side (*p* = 0.047 and *p* = 0.028, respectively). All mean differences, exact *p*-values and confidence intervals for the ANCOVAs between the FBTCS-Y, FBTCS-N, and Control groups are provided in Supplementary Tables 3, 4.

### 3.4. Drug resistant vs. drug responsive patients

Compared to the drug responsive group, the drug resistant group had higher mean TW-AD and TW-FA in the commissure anterior (CA) and this difference was significant (TW-AD: *p* = 0.045, partial *n*^2^ = 0.178, 95% CI [9.116E-07, 7.560E-05]; TW-FA: *p* = 0.039, partial *n*^2^ = 0.187, 95% CI [1.408E-03, 5.089E-02]). The tests of between subjects effects showed that as a covariate, drug resistance was only significant in the model for the TW-AD and TW-FA of the commissure anterior and did not change any prior results. Notably, significant TW-TM differences were not observed in the commissure anterior in the other grouping conditions.

## 4. Discussion

In this study, tract-specific TW-TM were obtained to quantify the white matter differences in patients with and without FBTCS, compared to controls.

Our study revealed patients with FBTCS had unexpected, marked alterations in specific subcortical tracts considered critical to seizure genesis. Our findings indicated that the type of inferred white matter disruption (i.e., altered TW-AD) taken in the context of the subcortical region, may be relevant to the seizure semiology observed in FBTCS. The region-specific alterations are likely a function of seizure pathology rather than whole-brain differences. The ensuing discussion focuses solely on the compelling results that could yield clinical utility. Of particular interest were the whole brain tracts, where significantly different mean TW-TM were observed between the patient subgroups. Readers may refer to the Supplementary Tables to further examine other results.

The first remarkable finding was the significant difference between the two patient subgroups in the TW-ADC and TW-AD of the inferior fronto-occipital fascicle (IFO), and striatal and thalamic occipital tracts (STOCC and TOCC), providing support for the second hypothesis. The thalamocortical circuit has been implicated as a critical mechanism for the genesis of FBTCS (43) by engagement of the striatum and corpus callosum. In patients with mesio-TLE, stereotactic EEG (SEEG) has shown the role of thalamocortical connections in seizure termination (44). Altered thalamic functional profiles have also been proposed as imaging biomarkers of active secondary generalization (45).

Although the inhibitory role of the basal ganglia has been demonstrated in TLE with FBTCS, our study shows it may be important in other focal epilepsies presenting with FBTCS. Mounting research using stereo-EEG, deep brain stimulation, and EEG/fMRI suggests the physiological rhythms in the basal ganglia as a “pacemaker” for ictal discharge throughout adjacent regions (12). Stereo-EEG demonstrated cortico-striatal synchronization (46), implicating the changing synchronization as a mechanism to control the duration of abnormal oscillations within the striatal-thalamo-cortical loop and for potential termination. Deep brain stimulation studies show that regulation of basal ganglia activity *via* thalamostriatal projections may mediate generalized seizures (47). Combined EEG/fMRI measurements showed the thalamocortical-striatal network could be involved in activation (thalamocortical), deactivation (striatal), and consequently termination of cortical discharge (48). Furthermore, the cortico-striato-thalamo-cerebellar network has been implicated as a prominent feature of FBTCS *via* increased connectivity of structural covariance in the striatum and thalamus (49). Here, we identified alterations in the TW-TM of specific tracts (inferior fronto-occipital fascicle, striato-occipital, and thalamo-occipital tracts) that may represent a predictive biomarker to differentiate between individuals who develop FBTCS vs. those who do not. The increased TW-ADC in conjunction with the increased TW-AD could infer white matter disruption significant enough to inhibit propagation of ictal discharge. Our finding in a new dataset using reproducible imaging techniques provides the field with further evidence congruent with existing research. This finding adds to the foundational knowledge of FBTCS and could guide researchers in investigating the specific circuits and networks involved in FBTCS.

Second, the significant difference observed between the FBTCS-N and control groups in three TW-TM (TW-ADC, TW-FA, and TW-RD) derived from the thalamo-prefrontal and thalamo-premotor tracts might be explained by premorbid connections in the subcortical structures. A recent application of magnetic resonance fingerprinting in normal appearing MRI scans from a focal epilepsy cohort revealed predominantly ipsilateral changes in the tissue properties of the thalamus and basal ganglia (50), suggesting both the impairment and vulnerability of subcortical structures in focal epilepsy. In TLE, propagation patterns of electrophysiological discharge are bolstered by the structural white matter pathways which reinforce ictal propagation (51), suggesting that premorbid white matter damage may halt the natural progression of ictal discharge. However, for those with less altered TPREF and TPREM tracts, such as in our FBTCS-Y group, the white matter fibre bundles may bear a closer resemblance to the control group. Here, ictal discharge events could gain enough momentum to override the inhibitory mechanisms and spread to the contralateral hemisphere.

Lastly, given the overall significant differences were observed between the FBTCS-N group and controls, the significantly higher TW-AD of the middle cerebellar peduncle in the FBTCS-Y group is noteworthy. Unlike the thalamic tracts, the middle cerebellar peduncle is cross-hemispheric, connecting the cerebellum to the pons, and is composed entirely of centripetal fibres. Previous work has shown the role of the middle cerebellar peduncle in generalized tonic-clonic seizures (52), potentiating it as a tract where injury may increase susceptibility to the development of a generalized seizure.

Although increased AD has been shown to result from white matter maturation, all groups were age-matched, suggesting the possibility that the higher TW-AD in the middle cerebellar peduncle of the FBTCS-Y group may be due to distinct premorbid FBTCS mechanisms. Since increased AD can also imply better organization of fibre structure, the TW-AD differences between the FBTCS-Y and FBTCS-N groups in the cerebellar region could be a feature of the FBTCS brain whereby those with FBTCS-Y have stronger premorbid fibre connections and the ability to sustain the intrahemispheric flow of ictal discharge. The middle cerebellar peduncle is presented as a compelling region of interest in post-surgical imaging, and future works could investigate this further.

It is noted that although the inferior cerebellar peduncle makes up the peduncle group and is anatomically adjacent to the superior and middle cerebellar peduncles, it is neither functionally important in information relay nor inhibition, which may explain the absence of significant, between groups difference in this tract. Finally, there was no significant relationship between lesion presence and seizure onset side, or lesion presence and FBTCS, reinforcing the conjecture that the tract-specific TW-TM differences between the patient subgroups were due to premorbid connections rather than the effects of seizure injury. Our findings promote the possibility that propagation of ictal discharge from the epileptogenic zone to the contralateral hemisphere could be inhibited by premorbid damage to critical information relay and anatomically relevant tracts.

Imaging studies of patients with epilepsy are traditionally limited by small sample sizes (19–21, 53), primarily due to challenges in recruitment. Our relatively small sample size was a limitation in our study, which prevented further patient subgroup analysis of the striatal and thalamus tracts. Nevertheless, our study presents to the field specific tracts that may play a role in FBTCS, reinforcing the value of imaging techniques such as TW-TM in demystifying the mechanisms involved in FBTCS. Though generalized seizures may appear similar in semiology to FBTCS, specific pathways and networks may be involved in FBTCS once the bi-hemispheric ictal propagation begins and is a critical topic for further research. There is the possibility that the seizure onset zone may have a role in the observed differences. However, in the current sample size, such analysis would be low powered. Future work with a larger sample size would be better equipped to investigate such queries.

In summary, we used a robust and reproducible model to obtain track-specific TW-TM to show region-specific white matter alterations in a subgroup of patients with FBTCS, compared to those with focal epilepsy alone, and control participants. Our findings provide mechanistic insights into how structural changes may impact the functional role of the thalamic and striatal regions in individuals with FBCTS. We highlight specific tracts (IFO, STOCC, TPREF, and TPREM) that may be involved in FBTCS. Our results lay the foundation for a better understanding of seizure propagation in FBCTS and offer potential biomarkers that can help explain disease progression and aid treatment.

## Data availability statement

The datasets generated and/or analyzed in the current study are not publicly available as they are from RPAH patients, and only individuals named on the approved ethics are authorized to access the data. However, de-identified data can be made available on reasonable request to the corresponding author, subject to approval from the relevant governing ethics entities.

## Ethics statement

The studies involving human participants were reviewed and approved by the RPAH Sydney Local Health District (RPAH-SLHD) Ethics Committee. Written informed consent for participation was not required for this study in accordance with the national legislation and the institutional requirements.

## Author contributions

CM: conceptualization, methodology, data curation, imaging analysis, statistical analysis, manuscript writing, and review and editing. AD'S: imaging and diffusion analysis pipeline implementation, data curation and analysis, and manuscript revision. RZ: imaging pipeline—data acquisition and Tractseg implementation. MB and OK: conceptualization and manuscript revision. AN: clinical data acquisition, clinical advisory, conceptualization, methodology, and manuscript revision. CW: conceptualization, methodology, data curation, imaging analysis, statistical analysis, and manuscript revision. All authors contributed to the article and approved the submitted version.

## Funding

This study received funding from UCB. The funder was not involved in the study design, collection, analysis, interpretation of data, the writing of this article or the decision to submit it for publication.

## Conflict of interest

The authors declare that the research was conducted in the absence of any commercial or financial relationships that could be construed as a potential conflict of interest.

## Publisher's note

All claims expressed in this article are solely those of the authors and do not necessarily represent those of their affiliated organizations, or those of the publisher, the editors and the reviewers. Any product that may be evaluated in this article, or claim that may be made by its manufacturer, is not guaranteed or endorsed by the publisher.

## References

[1] Park KJ Sharma G Kennedy JD Seyal M. Potentially high-risk cardiac arrhythmias with focal to bilateral tonic-clonic seizures and generalized tonic-clonic seizures are associated with the duration of periictal hypoxemia. Epilepsia. (2017) 58:2164–71. 10.1111/epi.13934 29105057

[2] Lawn ND Bamlet W Radhakrishnan K O'Brien P So EL. Injuries due to seizures in persons with epilepsy: a population-based study. Neurology. (2004) 63:1565–70. 10.1212/01.WNL.0000142991.14507.B5 15534237

[3] Sveinsson O Andersson T Carlsson S Tomson T. The incidence of SUDEP: a nationwide population-based cohort study. Neurology. (2017) 89:170–7. 10.1212/WNL.0000000000004094 28592455

[4] Blumenfeld H Westerveld M Ostroff RB Vanderhill SD Freeman J Necochea A. Selective frontal, parietal, and temporal networks in generalized seizures. Neuroimage. (2003) 19:1556–66. 10.1016/S1053-8119(03)00204-0 12948711

[5] Holmes MD Brown M Tucker DM. Are “generalized” seizures truly generalized? Evidence of localized mesial frontal and frontopolar discharges in absence. Epilepsia. (2004) 45:1568–79. 10.1111/j.0013-9580.2004.23204.x 15571515

[6] Schindler K Leung H Lehnertz K Elger CE. How generalised are secondarily “generalised” tonic-clonic seizures? J Neurol Neurosurg Psychiatry. (2007) 78:993–6. 10.1136/jnnp.2006.108753 17237141 PMC2117860

[7] Hwang K Bertolero MA Liu WB D'Esposito M. The human thalamus is an integrative hub for functional brain networks. J Neurosci. (2017) 37:5594–607. 10.1523/JNEUROSCI.0067-17.2017 28450543 PMC5469300

[8] Jones EG. The Thalamus. New York, NY: Springer (2012).

[9] Norden AD Blumenfeld H. The role of subcortical structures in human epilepsy. Epilepsy Behav. (2002) 3:219–31. 10.1016/S1525-5050(02)00029-X 12662601

[10] Blumenfeld H. The thalamus and seizures. Arch Neurol. (2002) 59:135–7. 10.1001/archneur.59.1.135 11790241

[11] Smith Y Bevan M Shink E Bolam JP. Microcircuitry of the direct and indirect pathways of the basal ganglia. Neuroscience. (1998) 86:353–87. 9881853 10.1016/s0306-4522(98)00004-9

[12] Rektor I Kuba R Brázdil M Chrastina J. Do the basal ganglia inhibit seizure activity in temporal lobe epilepsy? Epilepsy Behav. (2012) 25:56–9. 10.1016/j.yebeh.2012.04.125 22835431

[13] Feddersen B Remi J Kilian M Vercueil L Deransart C Depaulis A. Is ictal dystonia associated with an inhibitory effect on seizure propagation in focal epilepsies? Epilepsy Res. (2012) 99:274–80. 10.1016/j.eplepsyres.2011.12.007 22277599

[14] Uchida CGP Barsottini OGP Caboclo LOSF de Araújo Filho GM Centeno RS Junior HC. Does the patient's hand hold the key to preventing secondary generalization in mesial temporal lobe epilepsy? Epilepsy Res. (2013) 105:125–32. 10.1016/j.eplepsyres.2013.02.001 23490657

[15] Rektor I Kuba R Brázdil M. Interictal and ictal EEG activity in the basal ganglia: an SEEG study in patients with temporal lobe epilepsy. Epilepsia. (2002) 43:253–62. 10.1046/j.1528-1157.2002.28001.x 11906510

[16] Otte WM van Eijsden P Sander JW Duncan JS Dijkhuizen RM Braun KP. A meta-analysis of white matter changes in temporal lobe epilepsy as studied with diffusion tensor imaging. Epilepsia. (2012) 53:659–67. 10.1111/j.1528-1167.2012.03426.x 22379949

[17] Slinger G Sinke MR Braun KP Otte WM. White matter abnormalities at a regional and voxel level in focal and generalized epilepsy: a systematic review and meta-analysis. Neuroimage Clin. (2016) 12:902–9. 10.1016/j.nicl.2016.10.025 27882296 PMC5114611

[18] Campos BM Coan AC Beltramini GC Liu M Yassuda CL Ghizoni E. White matter abnormalities associate with type and localization of focal epileptogenic lesions. Epilepsia. (2015) 56:125–32. 10.1111/epi.12871 25545559

[19] Chiang S Levin HS Wilde E Haneef Z. White matter structural connectivity changes correlate with epilepsy duration in temporal lobe epilepsy. Epilepsy Res. (2016) 120:37–46. 10.1016/j.eplepsyres.2015.12.002 26709881 PMC4740226

[20] Lemkaddem A Daducci A Kunz N Lazeyras F Seeck M Thiran JP. Connectivity and tissue microstructural alterations in right and left temporal lobe epilepsy revealed by diffusion spectrum imaging. Neuroimage Clin. (2014) 5:349–58. 10.1016/j.nicl.2014.07.013 26236626 PMC4519999

[21] Sone D Sato N Kimura Y Watanabe Y Okazaki M Matsuda H. Brain morphological and microstructural features in cryptogenic late-onset temporal lobe epilepsy: a structural and diffusion MRI study. Neuroradiology. (2018) 60:635–41. 10.1007/s00234-018-2019-z 29654334

[22] Sinha N Peternell N Schroeder GM de Tisi J Vos SB Winston GP. Focal to bilateral tonic-clonic seizures are associated with widespread network abnormality in temporal lobe epilepsy. Epilepsia. (2021) 62:729–41. 10.1111/epi.16819 33476430 PMC8600951

[23] Willats L Raffelt D Smith RE Tournier JD Connelly A Calamante F. Quantification of track-weighted imaging (TWI): characterisation of within-subject reproducibility and between-subject variability. Neuroimage. (2014) 87:18–31. 10.1016/j.neuroimage.2013.11.016 24246491

[24] Pizzo F Roehri N Giusiano B Lagarde S Carron R Scavarda D. The ictal signature of thalamus and basal ganglia in focal epilepsy: a SEEG study. Neurology. (2021) 96:e280-e93. 10.1212/WNL.0000000000011003 33024023

[25] Hatton SN Huynh KH Bonilha L Abela E Alhusaini S Altmann A. White matter abnormalities across different epilepsy syndromes in adults: an ENIGMA-Epilepsy study. Brain. (2020) 143:2454–73. 10.1093/brain/awaa200 32814957 PMC7567169

[26] Wasserthal J Neher P Maier-Hein KH. TractSeg-Fast and accurate white matter tract segmentation. Neuroimage. (2018) 183:239–53. 10.1016/j.neuroimage.2018.07.070 30086412

[27] Andersson JL Sotiropoulos SN. An integrated approach to correction for off-resonance effects and subject movement in diffusion MR imaging. Neuroimage. (2016) 125:1063–78. 10.1016/j.neuroimage.2015.10.019 26481672 PMC4692656

[28] Fischl B. Freesurfer. Neuroimage. (2012) 62:774–81. 10.1016/j.neuroimage.2012.01.021 22248573 PMC3685476

[29] Smith R Skoch A Bajada CJ Caspers S Connelly A. Hybrid surface-volume segmentation for improved anatomically-constrained tractography. In: OHBM Annual Meeting. (2020). p. 1–5.

[30] Tournier JD Smith R Raffelt D Tabbara R Dhollander T Pietsch M. MRtrix3: A fast, flexible and open software framework for medical image processing and visualisation. Neuroimage. (2019) 202:116137. 10.1016/j.neuroimage.2019.116137 31473352

[31] Smith SM Jenkinson M Woolrich MW Beckmann CF Behrens TE Johansen-Berg H. Advances in functional and structural MR image analysis and implementation as FSL. Neuroimage. (2004) 23:S208–19. 10.1016/j.neuroimage.2004.07.051 15501092

[32] Tustison NJ Avants BB Cook PA Zheng Y Egan A Yushkevich PA. N4ITK: improved N3 bias correction. IEEE Trans Med Imaging. (2010) 29:1310–20. 10.1109/TMI.2010.2046908 20378467 PMC3071855

[33] Dhollander T Raffelt D Connelly A. Unsupervised 3-tissue response function estimation from single-shell or multi-shell diffusion MR data without a co-registered T1 image. In: ISMRM Workshop on Breaking the Barriers of Diffusion MRI. Lisbon: ISMRM (2016).

[34] Smith RE Tournier JD Calamante F Connelly A. Anatomically-constrained tractography: improved diffusion MRI streamlines tractography through effective use of anatomical information. Neuroimage. (2012) 62:1924–38. 10.1016/j.neuroimage.2012.06.005 22705374

[35] Tournier JD Calamante F Connelly A. Improved probabilistic streamlines tractography by 2nd order integration over fibre orientation distributions. In: Proceedings of the International Society for Magnetic Resonance in Medicine. New Jersey, NJ: John Wiley & Sons, Inc. (2010).

[36] Smith RE Tournier JD Calamante F Connelly A. SIFT2: Enabling dense quantitative assessment of brain white matter connectivity using streamlines tractography. Neuroimage. (2015) 119:338–51. 10.1016/j.neuroimage.2015.06.092 26163802

[37] Calamante F Tournier JD Jackson GD Connelly A. Track-density imaging (TDI): super-resolution white matter imaging using whole-brain track-density mapping. Neuroimage. (2010) 53:1233–43. 10.1016/j.neuroimage.2010.07.024 20643215

[38] Alexander AL Lee JE Lazar M Field AS. Diffusion tensor imaging of the brain. Neurotherapeutics. (2007) 4:316–29. 10.1016/j.nurt.2007.05.011 17599699 PMC2041910

[39] Calamante F. Track-weighted imaging methods: extracting information from a streamlines tractogram. Magnet Reson Mater Phys Biol Med. (2017) 30:317–35. 10.1007/s10334-017-0608-1 28181027

[40] Wilcox R. Robust ANCOVA, curvature, and the curse of dimensionality. J Modern Appl Stat Methods. (2019) 17:2. 10.22237/jmasm/1551906370

[41] Sinha N Johnson GW Davis KA Englot DJ. Integrating network neuroscience into epilepsy care: progress, barriers, and next steps. Epilepsy Curr. (2022) 0:1–7. 10.1177/15357597221101271 PMC9549227 36285209

[42] Lebel C Gee M Camicioli R Wieler M Martin W Beaulieu C. Diffusion tensor imaging of white matter tract evolution over the lifespan. Neuroimage. (2012) 60:340–352. 10.1016/j.neuroimage.2011.11.094 22178809

[43] Brodovskaya A Kapur J. Circuits generating secondarily generalized seizures. Epilepsy Behav. (2019) 101:106474. 10.1016/j.yebeh.2019.106474 31431400 PMC6944760

[44] Evangelista E Bénar C Bonini F Carron R Colombet B Régis J. Does the thalamo-cortical synchrony play a role in seizure termination? Front Neurol. (2015) 6:192. 10.3389/fneur.2015.00192 26388834 PMC4555023

[45] Caciagli L Allen LA He X Trimmel K Vos SB Centeno M. Thalamus and focal to bilateral seizures: a multiscale cognitive imaging study. Neurology. (2020) 95:e2427–41. 10.1212/WNL.0000000000010645 32847951 PMC7682917

[46] Aupy J Wendling F Taylor K Bulacio J Gonzalez-Martinez J Chauvel P. Cortico-striatal synchronization in human focal seizures. Brain. (2019) 142:1282–95. 10.1093/brain/awz062 30938430

[47] Velasco AL Velasco F Jiménez F Velasco M Castro G Carrillo-Ruiz JD. Neuromodulation of the centromedian thalamic nuclei in the treatment of generalized seizures and the improvement of the quality of life in patients with Lennox-Gastaut syndrome. Epilepsia. (2006) 47:1203–12. 10.1111/j.1528-1167.2006.00593.x 16886984

[48] Moeller F Siebner HR Wolff S Muhle H Boor R Granert O. Changes in activity of striato-thalamo-cortical network precede generalized spike wave discharges. NeuroImage. (2008) 39:1839–49. 10.1016/j.neuroimage.2007.10.058 18082429

[49] Xu Q Zhang Q Yang F Weng Y Xie X Hao J. Cortico-striato-thalamo-cerebellar networks of structural covariance underlying different epilepsy syndromes associated with generalized tonic-clonic seizures. Hum Brain Mapp. (2021) 42:1102–15. 10.1002/hbm.25279 33372704 PMC7856655

[50] Tang Y Su TY Choi JY Hu S Wang X Sakaie K. Characterizing thalamic and basal ganglia nuclei in medically intractable focal epilepsy by MR fingerprinting. Epilepsia. (2022) 63:1998–2010. 10.1111/epi.17318 35661353

[51] Gleichgerrcht E Greenblatt AS Kellermann TS Rowland N Vandergrift WA Edwards J. Patterns of seizure spread in temporal lobe epilepsy are associated with distinct white matter tracts. Epilepsy Res. (2021). 171:106571. 10.1016/j.eplepsyres.2021.106571 33582534 PMC7981262

[52] Jiang S Li X Li Z Chang X Chen Y Huang Y. Cerebello-cerebral connectivity in idiopathic generalized epilepsy. Eur Radiol. (2020) 30:3924–33. 10.1007/s00330-020-06674-3 32125514

[53] Liu M Chen Z Beaulieu C Gross DW. Disrupted anatomic white matter network in left mesial temporal lobe epilepsy. Epilepsia. (2014) 55:674–82. 10.1111/epi.12581 24650167

