# Supplementary material for: White matter alterations in focal to bilateral tonic-clonic seizures
Source: Front Neurol. 2022 Sep 14;13:972590. doi: 10.3389/fneur.2022.972590 (PMC9515421; doi:10.3389/fneur.2022.972590)
Supplement: Supplementary file 1 [file Table_1.pdf]

Supplementary Table 1. Group differences between the whole brain, mean TW-TM of each tract in the “All patients” group compared to controls

	**TW-ADC**					**TW-FA**					**TW-RD**					**TW-AD**				
**Tracts**	**Mean Difference**	***p***	**η_p^2**	**95% CI - Lower**	**95% CI - Upper**	**Mean Difference**	***p***	**η_p^2**	**95% CI - Lower**	**95% CI - Upper**	**Mean Difference**	***p***	**η_p^2**	**95% CI - Lower**	**95% CI - Upper**	**Mean Difference**	***p***	**η_p^2**	**95% CI - Lower**	**95% CI - Upper**
Anterior thalamic radiation (ATR)	**1.687E-5***	**0.013**	**0.143**	**3.685E-06**	**3.005E-05**	-0.007	0.114	0.061	-0.016	0.002	**1.826E-5***	**0.008**	**0.165**	**5.150E-06**	**3.137E-05**	1.408E-05	0.103	0.065	-2.974E-06	3.114E-05
Superior thalamic radiation (STR)	**1.727E-5***	**0.010**	**0.156**	**4.427E-06**	**3.012E-05**	-0.010	0.120	0.059	-0.024	0.003	**1.956E-5***	**0.008**	**0.161**	**5.312E-06**	**3.381E-05**	1.270E-05	0.207	0.039	-7.331E-06	3.273E-05
Corticospinal (CST)	**1.106E-5***	**0.044**	**0.098**	**3.328E-07**	**2.178E-05**	-0.007	0.187	0.043	-0.018	0.004	**1.239E-5***	**0.043**	**0.099**	**4.347E-07**	**2.435E-05**	8.391E-06	0.300	0.027	-7.769E-06	2.455E-05
Fronto pontine (FPT)	**1.277E-5***	**0.035**	**0.106**	**9.409E-07**	**2.459E-05**	-0.006	0.155	0.050	-0.016	0.003	**1.416E-5***	**0.026**	**0.117**	**1.753E-06**	**2.656E-05**	9.974E-06	0.203	0.040	-5.595E-06	2.554E-05
Parieto-occipital pontine (POPT)	1.003E-05	0.078	0.076	-1.175E-06	2.123E-05	-0.008	0.144	0.053	-0.018	0.003	1.232E-05	0.052	0.091	-1.193E-07	2.476E-05	5.451E-06	0.463	0.014	-9.428E-06	2.033E-05
Inferior cerebellar peduncle (ICP)	6.203E-06	0.268	0.031	-4.957E-06	1.736E-05	0.004	0.502	0.011	-0.008	0.017	3.340E-06	0.604	0.007	-9.584E-06	1.626E-05	1.193E-05	0.100	0.066	-2.387E-06	2.625E-05
Middle cerebellar peduncle (MCP)	6.455E-06	0.318	0.025	-6.457E-06	1.937E-05	0.011	0.130	0.056	-0.003	0.026	-1.968E-07	0.979	0.000	-1.519E-05	1.479E-05	**1.976E-5***	**0.033**	**0.109**	**1.668E-06**	**3.785E-05**
Superior cerebellar peduncle (SCP)	**1.234E-5***	**0.026**	**0.117**	**1.528E-06**	**2.316E-05**	0.004	0.441	0.015	-0.007	0.016	7.848E-06	0.142	0.053	-2.731E-06	1.843E-05	**2.1331E-5***	**0.032**	**0.110**	**1.963E-06**	**4.070E-05**
Inferior fronto occipital (IFO)	**1.446E-5***	**0.042**	**0.099**	**5.233E-07**	**2.839E-05**	-0.008	0.154	0.050	-0.018	0.003	**1.631E-5***	**0.031**	**0.111**	**1.557E-06**	**3.105E-05**	1.076E-05	0.190	0.043	-5.546E-06	2.706E-05
ST_ Fronto orbital (STFO)	1.374E-05	0.057	0.088	-4.027E-07	2.789E-05	-0.007	0.158	0.049	-0.017	0.003	**1.514E-5***	**0.043**	**0.099**	**5.312E-07**	**2.974E-05**	1.095E-05	0.211	0.039	-6.455E-06	2.836E-05
ST_Occipital (STOCC)	**1.554E-5***	**0.046**	**0.096**	**2.660E-07**	**3.082E-05**	-0.007	0.247	0.033	-0.019	0.005	**1.706E-5***	**0.044**	**0.098**	**5.097E-07**	**3.362E-05**	1.250E-05	0.162	0.048	-5.226E-06	3.022E-05
ST_Parietal (STPAR)	1.166E-05	0.077	0.076	-1.335E-06	2.466E-05	-0.010	0.093	0.069	-0.021	0.002	**1.460E-5***	**0.045**	**0.097**	**3.390E-07**	**2.886E-05**	5.784E-06	0.455	0.014	-9.705E-06	2.127E-05
ST_Postcentral (STPOSTC)	**1.344E-5***	**0.028**	**0.115**	**1.505E-06**	**2.537E-05**	-0.009	0.124	0.058	-0.021	0.003	**1.498E-5***	**0.026**	**0.118**	**1.887E-06**	**2.807E-05**	1.036E-05	0.216	0.038	-6.291E-06	2.701E-05
ST_Precentral (STPREC)	**1.599E-5***	**0.014**	**0.141**	**3.384E-06**	**2.860E-05**	-0.010	0.081	0.074	-0.021	0.001	**1.754E-5***	**0.013**	**0.146**	**3.970E-06**	**3.112E-05**	1.288E-05	0.122	0.059	-3.576E-06	2.934E-05
ST_Prefrontal (STPREF)	**1.532E-5***	**0.026**	**0.118**	**1.948E-06**	**2.869E-05**	-0.008	0.094	0.069	-0.017	0.001	**1.691E-5***	**0.017**	**0.134**	**3.183E-06**	**3.064E-05**	1.213E-05	0.134	0.055	-3.882E-06	2.815E-05
ST_Premotor (STPREM)	**1.575E-5***	**0.024**	**0.122**	**2.218E-06**	**2.928E-05**	-0.007	0.126	0.057	-0.017	0.002	**1.638E-5***	**0.020**	**0.127**	**2.679E-06**	**3.008E-05**	1.448E-05	0.088	0.071	-2.263E-06	3.123E-05
T_Occipital (TOCC)	**1.790E-5***	**0.027**	**0.117**	**2.167E-06**	**3.363E-05**	-0.008	0.166	0.047	-0.020	0.004	**1.989E-5***	**0.022**	**0.125**	**3.064E-06**	**3.672E-05**	1.392E-05	0.131	0.056	-4.334E-06	3.217E-05
T_Postcentral (TPOSTC)	**1.360E-5***	**0.019**	**0.131**	**2.387E-06**	**2.480E-05**	-0.009	0.160	0.049	-0.021	0.004	**1.487E-5***	**0.022**	**0.124**	**2.215E-06**	**2.753E-05**	1.104E-05	0.192	0.042	-5.790E-06	2.787E-05
T_Precentral (TPREC)	**1.688E-5***	**0.008**	**0.162**	**4.623E-06**	**2.913E-05**	-0.010	0.075	0.077	-0.022	0.001	**1.864E-5***	**0.008**	**0.164**	**5.213E-06**	**3.207E-05**	1.335E-05	0.112	0.062	-3.231E-06	2.993E-05
T_Prefrontal (TPREF)	**1.686E-5***	**0.014**	**0.143**	**3.647E-06**	**3.008E-05**	-0.008	0.091	0.070	-0.017	0.001	**1.839E-5***	**0.009**	**0.159**	**4.870E-06**	**3.191E-05**	1.381E-05	0.094	0.069	-2.460E-06	3.008E-05
T_Premotor (TPREM)	**1.728E-5***	**0.009**	**0.157**	**4.463E-06**	**3.009E-05**	-0.007	0.143	0.053	-0.017	0.003	**1.773E-5***	**0.009**	**0.160**	**4.750E-06**	**3.071E-05**	1.637E-05	0.055	0.089	-4.007E-07	3.314E-05
Uncinate fascicle (UF)	1.324E-05	0.066	0.082	-9.223E-07	2.741E-05	**-.012***	**0.023**	**0.123**	**-0.022**	**-0.002**	**1.671E-5***	**0.020**	**0.127**	**2.729E-06**	**3.070E-05**	6.286E-06	0.509	0.011	-1.277E-05	2.534E-05
Commissure anterior (CA)	8.247E-06	0.253	0.033	-6.128E-06	2.262E-05	-0.004	0.552	0.009	-0.018	0.010	8.290E-06	0.309	0.026	-7.986E-06	2.457E-05	8.160E-06	0.416	0.017	-1.191E-05	2.823E-05
Corpus callosum (CC)	1.287E-05	0.057	0.088	-3.895E-07	2.614E-05	-0.009	0.065	0.083	-0.020	0.001	**1.584E-5***	**0.030**	**0.112**	**1.613E-06**	**3.007E-05**	6.936E-06	0.349	0.022	-7.867E-06	2.174E-05

Key: Mean difference: the mean difference of the "all patients" group minus the control group, based on the univariate ANCOVA estimated marginal means. Positive mean difference values indicate the "all patients" group had a higher average TW-TM than the control group, negative values indicate the control group had a higher TW-TM than "all patients". The asterisk "*" indicates a significant difference observed in the Bonferroni corrected univariate ANCOVA; the "p" value indicates whether the mean difference was significant at 0.05; " η_p^2" is the partial eta squared statistic.

Supplementary Table 2. Group differences between the mean TW-TM of each left and right tract in the "All patients" group compared to controls

	TW-ADC									
	Left					Right				
Tract	Mean Difference	*p*	η_p^2	95% CI - Lower	95% CI - Upper	Mean Difference	*p*	η_p^2	95% CI - Lower	95% CI - Upper
Anterior thalamic radiation (ATR)	1.552E-5*	0.022	0.124	2.311E-06	2.874E-05	1.832E-5*	0.009	0.159	4.844E-06	3.180E-05
Superior thalamic radiation (STR)	1.828E-5*	0.008	0.164	5.085E-06	3.147E-05	1.648E-5*	0.018	0.132	2.946E-06	3.001E-05
Corticospinal (CST)	1.143E-5*	0.043	0.098	3.662E-07	2.249E-05	1.074E-05	0.059	0.086	-4.379E-07	2.191E-05
Fronto pontine (FPT)	1.189E-05	0.053	0.091	-1.414E-07	2.392E-05	1.370E-5*	0.025	0.120	1.828E-06	2.556E-05
Parieto-occipital pontine (POPT)	1.122E-05	0.055	0.089	-2.591E-07	2.270E-05	9.192E-06	0.142	0.053	-3.223E-06	2.161E-05
Inferior cerebellar peduncle (ICP)	4.252E-06	0.438	0.015	-6.714E-06	1.522E-05	8.313E-06	0.185	0.043	-4.149E-06	2.077E-05
Superior cerebellar peduncle (SCP)	1.248E-5*	0.025	0.120	1.662E-06	2.331E-05	1.231E-5*	0.034	0.107	9.515E-07	2.367E-05
Inferior fronto occipital (IFO)	1.405E-05	0.054	0.090	-2.611E-07	2.835E-05	1.534E-5*	0.042	0.099	5.870E-07	3.009E-05
ST_ Fronto orbital (STFO)	1.256E-05	0.078	0.076	-1.485E-06	2.662E-05	1.541E-5*	0.044	0.097	4.107E-07	3.040E-05
ST_Occipital (STOCC)	1.462E-05	0.091	0.070	-2.437E-06	3.167E-05	1.791E-5*	0.035	0.106	1.290E-06	3.454E-05
ST_Parietal (STPAR)	1.272E-05	0.058	0.087	-4.549E-07	2.590E-05	1.099E-05	0.122	0.059	-3.049E-06	2.502E-05
ST_Postcentral (STPOSTC)	1.555E-5*	0.011	0.152	3.812E-06	2.728E-05	1.112E-05	0.100	0.066	-2.208E-06	2.445E-05
ST_Precentral (STPREC)	1.738E-5*	0.009	0.157	4.507E-06	3.026E-05	1.450E-5*	0.034	0.107	1.125E-06	2.789E-05
ST_Prefrontal (STPREF)	1.428E-5*	0.035	0.106	1.019E-06	2.755E-05	1.647E-5*	0.020	0.128	2.741E-06	3.019E-05
ST_Premotor (STPREM)	1.580E-5*	0.027	0.116	1.885E-06	2.972E-05	1.574E-5*	0.025	0.119	2.067E-06	2.942E-05
T_Occipital (TOCC)	1.697E-05	0.072	0.079	-1.581E-06	3.552E-05	2.075E-5*	0.020	0.128	3.470E-06	3.802E-05
T_Postcentral (TPOSTC)	1.551E-5*	0.007	0.167	4.434E-06	2.659E-05	1.165E-05	0.071	0.079	-1.057E-06	2.436E-05
T_Precentral (TPREC)	1.756E-5*	0.007	0.167	5.042E-06	3.008E-05	1.624E-5*	0.016	0.138	3.252E-06	2.922E-05
T_Prefrontal (TPREF)	1.589E-5*	0.020	0.128	2.660E-06	2.911E-05	1.795E-5*	0.011	0.153	4.437E-06	3.146E-05
T_Premotor (TPREM)	1.670E-5*	0.016	0.136	3.276E-06	3.012E-05	1.807E-5*	0.007	0.170	5.304E-06	3.084E-05
Uncinate fascicle (UF)	1.386E-5*	0.045	0.097	3.462E-07	2.738E-05	1.370E-05	0.081	0.074	-1.770E-06	2.918E-05

	TW-FA									
	Left					Right				
Tract	Mean Difference	*p*	η_p^2	95% CI - Lower	95% CI - Upper	Mean Difference	*p*	η_p^2	95% CI - Lower	95% CI - Upper
Anterior thalamic radiation (ATR)	-0.006	0.246	0.034	-0.015	0.004	-.009*	0.044	0.097	-0.018	0.000
Superior thalamic radiation (STR)	-0.013	0.066	0.082	-0.026	0.001	-0.008	0.218	0.038	-0.022	0.005
Corticospinal (CST)	-0.007	0.211	0.039	-0.018	0.004	-0.007	0.183	0.044	-0.018	0.003
Fronto pontine (FPT)	-0.006	0.175	0.045	-0.015	0.003	-0.007	0.144	0.053	-0.016	0.002
Parieto-occipital pontine (POPT)	-0.009	0.090	0.070	-0.020	0.002	-0.006	0.248	0.033	-0.017	0.004
Inferior cerebellar peduncle (ICP)	0.005	0.484	0.012	-0.009	0.018	0.003	0.561	0.009	-0.008	0.015
Superior cerebellar peduncle (SCP)	0.005	0.437	0.015	-0.007	0.017	0.004	0.467	0.013	-0.007	0.015
Inferior fronto occipital (IFO)	-0.008	0.171	0.046	-0.019	0.003	-0.008	0.157	0.049	-0.019	0.003
ST_ Fronto orbital (STFO)	-0.006	0.297	0.027	-0.016	0.005	-0.009	0.063	0.084	-0.019	0.001
ST_Occipital (STOCC)	-0.010	0.140	0.054	-0.023	0.003	-0.004	0.503	0.011	-0.017	0.008
ST_Parietal (STPAR)	-0.011	0.056	0.088	-0.022	0.000	-0.008	0.155	0.050	-0.020	0.003
ST_Postcentral (STPOSTC)	-0.011	0.061	0.085	-0.022	0.001	-0.007	0.251	0.033	-0.019	0.005
ST_Precentral (STPREC)	-0.011	0.055	0.089	-0.022	0.000	-0.009	0.138	0.054	-0.020	0.003
ST_Prefrontal (STPREF)	-0.007	0.130	0.056	-0.017	0.002	-0.009	0.069	0.080	-0.018	0.001
ST_Premotor (STPREM)	-0.007	0.152	0.051	-0.017	0.003	-0.008	0.116	0.061	-0.018	0.002
T_Occipital (TOCC)	-0.011	0.070	0.080	-0.024	0.001	-0.005	0.461	0.014	-0.017	0.008
T_Postcentral (TPOSTC)	-0.011	0.061	0.085	-0.023	0.001	-0.006	0.378	0.020	-0.018	0.007
T_Precentral (TPREC)	-.012*	0.050	0.093	-0.023	0.000	-0.009	0.131	0.056	-0.021	0.003
T_Prefrontal (TPREF)	-0.008	0.112	0.062	-0.017	0.002	-0.008	0.078	0.076	-0.018	0.001
T_Premotor (TPREM)	-0.006	0.181	0.044	-0.016	0.003	-0.008	0.123	0.058	-0.018	0.002
Uncinate fascicle (UF)	-0.010	0.063	0.084	-0.021	0.001	-.014*	0.009	0.159	-0.024	-0.004

TW-RD

Tract	Left Mean Difference	*p*	η_p^2	95% CI - Lower	95% CI - Upper	Right Mean Difference	*p*	η_p^2	95% CI - Lower	95% CI - Upper
Anterior thalamic radiation (ATR)	**1.607E-5***	**0.018**	**0.133**	**2.939E-06**	**2.921E-05**	**2.066E-5***	**0.003**	**0.194**	**7.222E-06**	**3.411E-05**
Superior thalamic radiation (STR)	**2.142E-5***	**0.005**	**0.182**	**6.919E-06**	**3.592E-05**	**1.788E-5***	**0.018**	**0.132**	**3.236E-06**	**3.253E-05**
Corticospinal (CST)	**1.266E-5***	**0.046**	**0.096**	**2.258E-07**	**2.510E-05**	1.211E-05	0.051	0.092	-3.252E-08	2.426E-05
Fronto pontine (FPT)	**1.324E-5***	**0.039**	**0.102**	**6.835E-07**	**2.579E-05**	**1.513E-5***	**0.019**	**0.130**	**2.624E-06**	**2.765E-05**
Parieto-occipital pontine (POPT)	**1.405E-5***	**0.031**	**0.112**	**1.387E-06**	**2.672E-05**	1.070E-05	0.105	0.064	-2.341E-06	2.375E-05
Inferior cerebellar peduncle (ICP)	1.431E-06	0.828	0.001	-1.182E-05	1.468E-05	5.549E-06	0.413	0.017	-7.996E-06	1.909E-05
Superior cerebellar peduncle (SCP)	7.733E-06	0.151	0.051	-2.944E-06	1.841E-05	8.062E-06	0.151	0.051	-3.071E-06	1.920E-05
Inferior fronto occipital (IFO)	**1.595E-5***	**0.040**	**0.101**	**7.623E-07**	**3.114E-05**	**1.708E-5***	**0.031**	**0.111**	**1.618E-06**	**3.253E-05**
ST_ Fronto orbital (STFO)	1.324E-05	0.072	0.078	-1.264E-06	2.775E-05	**1.768E-5***	**0.024**	**0.120**	**2.397E-06**	**3.295E-05**
ST_Occipital (STOCC)	1.769E-05	0.050	0.092	-3.867E-08	3.542E-05	1.731E-05	0.053	0.091	-2.213E-07	3.485E-05
ST_Parietal (STPAR)	**1.604E-5***	**0.029**	**0.114**	**1.737E-06**	**3.035E-05**	1.346E-05	0.080	0.075	-1.667E-06	2.858E-05
ST_Postcentral (STPOSTC)	**1.753E-5***	**0.008**	**0.162**	**4.798E-06**	**3.026E-05**	1.217E-05	0.096	0.068	-2.237E-06	2.657E-05
ST_Precentral (STPREC)	**1.906E-5***	**0.008**	**0.164**	**5.312E-06**	**3.281E-05**	**1.589E-5***	**0.031**	**0.111**	**1.493E-06**	**3.028E-05**
ST_Prefrontal (STPREF)	**1.553E-5***	**0.027**	**0.117**	**1.902E-06**	**2.917E-05**	**1.842E-5***	**0.012**	**0.149**	**4.364E-06**	**3.248E-05**
ST_Premotor (STPREM)	**1.578E-5***	**0.028**	**0.115**	**1.793E-06**	**2.977E-05**	**1.717E-5***	**0.018**	**0.132**	**3.098E-06**	**3.124E-05**
T_Occipital (TOCC)	**2.077E-5***	**0.030**	**0.113**	**2.137E-06**	**3.941E-05**	**2.017E-5***	**0.031**	**0.111**	**1.974E-06**	**3.837E-05**
T_Postcentral (TPOSTC)	**1.770E-5***	**0.006**	**0.174**	**5.393E-06**	**3.001E-05**	1.184E-05	0.095	0.068	-2.139E-06	2.582E-05
T_Precentral (TPREC)	**1.971E-5***	**0.006**	**0.177**	**6.112E-06**	**3.331E-05**	**1.752E-5***	**0.016**	**0.136**	**3.410E-06**	**3.163E-05**
T_Prefrontal (TPREF)	**1.723E-5***	**0.014**	**0.143**	**3.725E-06**	**3.073E-05**	**1.967E-5***	**0.007**	**0.171**	**5.825E-06**	**3.352E-05**
T_Premotor (TPREM)	**1.651E-5***	**0.016**	**0.137**	**3.246E-06**	**2.978E-05**	**1.920E-5***	**0.006**	**0.175**	**5.856E-06**	**3.254E-05**
Uncinate fascicle (UF)	**1.638E-5***	**0.021**	**0.126**	**2.611E-06**	**3.015E-05**	**1.819E-5***	**0.020**	**0.129**	**3.064E-06**	**3.331E-05**

TW-AD

Tract	Left Mean Difference	*p*	η_p^2	95% CI - Lower	95% CI - Upper	Right Mean Difference	*p*	η_p^2	95% CI - Lower	95% CI - Upper
Anterior thalamic radiation (ATR)	1.442E-05	0.102	0.066	-2.970E-06	3.182E-05	1.364E-05	0.117	0.060	-3.560E-06	3.083E-05
Superior thalamic radiation (STR)	1.199E-05	0.264	0.031	-9.405E-06	3.339E-05	1.368E-05	0.191	0.042	-7.095E-06	3.445E-05
Corticospinal (CST)	8.954E-06	0.305	0.026	-8.465E-06	2.637E-05	7.983E-06	0.322	0.025	-8.094E-06	2.406E-05
Fronto pontine (FPT)	9.196E-06	0.260	0.032	-7.086E-06	2.548E-05	1.082E-05	0.158	0.049	-4.363E-06	2.600E-05
Parieto-occipital pontine (POPT)	5.559E-06	0.485	0.012	-1.037E-05	2.149E-05	6.166E-06	0.457	0.014	-1.041E-05	2.274E-05
Inferior cerebellar peduncle (ICP)	9.894E-06	0.182	0.044	-4.819E-06	2.461E-05	1.384E-05	0.073	0.078	-1.328E-06	2.901E-05
Superior cerebellar peduncle (SCP)	**2.199E-5***	**0.035**	**0.106**	**1.602E-06**	**4.237E-05**	**2.081E-5***	**0.033**	**0.109**	**1.803E-06**	**3.981E-05**
Inferior fronto occipital (IFO)	1.024E-05	0.235	0.035	-6.926E-06	2.740E-05	1.187E-05	0.169	0.047	-5.246E-06	2.899E-05
ST_ Fronto orbital (STFO)	1.121E-05	0.209	0.039	-6.552E-06	2.898E-05	1.087E-05	0.241	0.034	-7.582E-06	2.932E-05
ST_Occipital (STOCC)	8.476E-06	0.446	0.015	-1.376E-05	3.072E-05	1.911E-05	0.054	0.089	-3.806E-07	3.860E-05
ST_Parietal (STPAR)	6.083E-06	0.454	0.014	-1.017E-05	2.234E-05	6.046E-06	0.466	0.013	-1.056E-05	2.265E-05
ST_Postcentral (STPOSTC)	1.158E-05	0.175	0.045	-5.381E-06	2.855E-05	9.026E-06	0.316	0.025	-8.938E-06	2.699E-05
ST_Precentral (STPREC)	1.403E-05	0.108	0.063	-3.200E-06	3.125E-05	1.174E-05	0.170	0.047	-5.231E-06	2.871E-05
ST_Prefrontal (STPREF)	1.178E-05	0.148	0.052	-4.370E-06	2.794E-05	1.255E-05	0.127	0.057	-3.722E-06	2.883E-05
ST_Premotor (STPREM)	1.585E-05	0.078	0.076	-1.850E-06	3.355E-05	1.289E-05	0.118	0.060	-3.437E-06	2.922E-05
T_Occipital (TOCC)	9.362E-06	0.431	0.016	-1.442E-05	3.315E-05	**2.189E-5***	**0.032**	**0.110**	**1.946E-06**	**4.184E-05**
T_Postcentral (TPOSTC)	1.114E-05	0.196	0.041	-5.974E-06	2.825E-05	1.128E-05	0.226	0.037	-7.237E-06	2.979E-05
T_Precentral (TPREC)	1.325E-05	0.135	0.055	-4.290E-06	3.080E-05	1.367E-05	0.112	0.062	-3.322E-06	3.067E-05
T_Prefrontal (TPREF)	1.321E-05	0.117	0.060	-3.441E-06	2.985E-05	1.450E-05	0.080	0.075	-1.789E-06	3.078E-05
T_Premotor (TPREM)	1.707E-05	0.065	0.083	-1.093E-06	3.524E-05	1.582E-05	0.052	0.091	-1.420E-07	3.177E-05
Uncinate fascicle (UF)	8.823E-06	0.340	0.023	-9.655E-06	2.730E-05	4.735E-06	0.644	0.005	-1.585E-05	2.532E-05

Key: Mean difference: the mean difference of the "all patients" group minus the control group, based on the univariate ANCOVA estimated marginal means. Positive mean difference values indicate the "all patients" group had a higher average TW-TM than the controls, negative values indicate the control group had a higher average TW-TM than "all patients". The asterisk "*" indicates a significant difference observed in the Bonferroni corrected univariate ANCOVA; the "p" value indicates whether the mean difference was significant at 0.05; "η_p^2" is the partial eta squared statistic.

Supplementary Table 3. Group differences between the whole brain, mean TW-TM of each tract in the FBTCS-N, FBTCS-Y and control groups.

			TW-ADC				TW-FA				TW-RD				TW-AD			
**Tracts**	**Group 1**	**Group 2**	**Mean Difference**	***p***	**95% CI Lower Bound**	**95% CI Upper Bound**	**Mean Difference**	***p***	**95% CI Lower Bound**	**95% CI Upper Bound**	**Mean Difference**	***p***	**95% CI Lower Bound**	**95% CI Upper Bound**	**Mean Difference**	***p***	**95% CI Lower Bound**	**95% CI Upper Bound**
Anterior thalamic radiation (ATR)	FBTCS-N	FBTCS-Y	1.775E-05	0.214	-6.225E-06	4.173E-05	-0.010	0.397	-0.027	0.006	1.835E-05	0.184	-5.481E-06	4.218E-05	1.656E-05	0.576	-1.465E-05	4.776E-05
		HC	**2.913E-5**[*]	**0.011**	**5.478E-06**	**5.278E-05**	-0.016	0.068	-0.032	0.001	**3.150E-5**[*]	**0.005**	**7.990E-06**	**5.501E-05**	2.439E-05	0.163	-6.387E-06	5.517E-05
	HC	FBTCS-Y	-1.138E-05	0.339	-2.893E-05	6.180E-06	0.005	0.838	-0.007	0.017	-1.315E-05	0.200	-3.060E-05	4.304E-06	-7.838E-06	1.000	-3.069E-05	1.501E-05
Superior thalamic radiation (STR)	FBTCS-N	FBTCS-Y	1.001E-05	0.913	-1.407E-05	3.409E-05	-0.011	0.761	-0.036	0.013	1.170E-05	0.838	-1.501E-05	3.841E-05	6.627E-06	1.000	-3.099E-05	4.425E-05
		HC	**2.408E-5**[*]	**0.046**	**3.239E-07**	**4.783E-05**	-0.019	0.156	-0.043	0.005	**2.814E-5**[*]	**0.033**	**1.792E-06**	**5.449E-05**	1.595E-05	0.865	-2.115E-05	5.306E-05
	HC	FBTCS-Y	-1.407E-05	0.159	-3.170E-05	3.565E-06	0.008	0.811	-0.010	0.026	-1.644E-05	0.126	-3.599E-05	3.120E-06	-9.326E-06	1.000	-3.687E-05	1.822E-05
Corticospinal (CST)	FBTCS-N	FBTCS-Y	1.355E-05	0.274	-6.042E-06	3.314E-05	-0.008	0.908	-0.028	0.012	1.301E-05	0.447	-9.112E-06	3.514E-05	1.463E-05	0.655	-1.464E-05	4.389E-05
		HC	**2.033E-5**[*]	**0.036**	**1.007E-06**	**3.966E-05**	-0.014	0.232	-0.034	0.005	**2.203E-5**[*]	**0.047**	**2.044E-07**	**4.385E-05**	1.695E-05	0.449	-1.192E-05	4.581E-05
	HC	FBTCS-Y	-6.782E-06	0.731	-2.113E-05	7.563E-06	0.006	0.945	-0.009	0.020	-9.015E-06	0.514	-2.521E-05	7.184E-06	-2.320E-06	1.000	-2.374E-05	1.911E-05
Fronto pontine (FPT)	FBTCS-N	FBTCS-Y	1.689E-05	0.166	-4.496E-06	3.828E-05	-0.010	0.360	-0.027	0.006	1.727E-05	0.188	-5.288E-06	3.983E-05	1.613E-05	0.483	-1.213E-05	4.438E-05
		HC	**2.446E-5**[*]	**0.018**	**3.364E-06**	**4.555E-05**	-0.015	0.079	-0.031	0.001	**2.671E-5**[*]	**0.014**	**4.459E-06**	**4.896E-05**	1.995E-05	0.243	-7.924E-06	4.782E-05
	HC	FBTCS-Y	-7.568E-06	0.701	-2.323E-05	8.090E-06	0.005	1.000	-0.007	0.016	-9.440E-06	0.481	-2.596E-05	7.076E-06	-3.823E-06	1.000	-2.451E-05	1.687E-05
Parieto-occipital pontine (POPT)	FBTCS-N	FBTCS-Y	1.757E-05	0.105	-2.540E-06	3.769E-05	-0.009	0.812	-0.028	0.011	1.611E-05	0.247	-6.502E-06	3.871E-05	2.051E-05	0.179	-5.945E-06	4.696E-05
		HC	**2.263E-5**[*]	**0.021**	**2.788E-06**	**4.246E-05**	-0.015	0.160	-0.034	0.004	**2.461E-5**[*]	**0.026**	**2.310E-06**	**4.691E-05**	1.866E-05	0.244	-7.432E-06	4.475E-05
	HC	FBTCS-Y	-5.053E-06	1.000	-1.978E-05	9.672E-06	0.007	0.760	-0.008	0.021	-8.504E-06	0.618	-2.506E-05	8.049E-06	1.847E-06	1.000	-1.752E-05	2.122E-05
Inferior cerebellar peduncle (ICP)	FBTCS-N	FBTCS-Y	7.696E-06	1.000	-1.220E-05	2.759E-05	-0.017	0.183	-0.039	0.005	1.349E-05	0.426	-9.049E-06	3.603E-05	-3.897E-06	1.000	-3.085E-05	2.305E-05
		HC	1.350E-05	0.279	-6.123E-06	3.313E-05	-0.010	0.847	-0.031	0.012	1.518E-05	0.286	-7.051E-06	3.742E-05	1.015E-05	1.000	-1.643E-05	3.673E-05
	HC	FBTCS-Y	-5.809E-06	0.973	-2.038E-05	8.760E-06	-0.008	0.750	-0.024	0.009	-1.692E-06	1.000	-1.820E-05	1.481E-05	-1.405E-05	0.248	-3.378E-05	5.687E-06
Middle cerebellar peduncle (MCP)	FBTCS-N	FBTCS-Y	-8.252E-07	1.000	-2.447E-05	2.282E-05	-0.021	0.148	-0.046	0.005	8.363E-06	1.000	-1.821E-05	3.494E-05	-1.920E-05	0.480	-5.276E-05	1.436E-05
		HC	7.628E-06	1.000	-1.569E-05	3.095E-05	-0.006	1.000	-0.031	0.020	8.389E-06	1.000	-1.782E-05	3.460E-05	6.106E-06	1.000	-2.699E-05	3.921E-05
	HC	FBTCS-Y	-8.454E-06	0.686	-2.576E-05	8.858E-06	-0.015	0.154	-0.034	0.004	-2.659E-08	1.000	-1.948E-05	1.943E-05	**-2.531E-5**[*]	**0.042**	**-4.988E-05**	**-7.365E-07**
Superior cerebellar peduncle (SCP)	FBTCS-N	FBTCS-Y	3.882E-06	1.000	-1.655E-05	2.431E-05	-0.019	0.072	-0.039	0.001	1.157E-05	0.410	-7.490E-06	3.064E-05	-1.150E-05	1.000	-4.800E-05	2.500E-05
		HC	1.569E-05	0.176	-4.458E-06	3.584E-05	-0.010	0.600	-0.030	0.009	1.735E-05	0.079	-1.455E-06	3.615E-05	1.238E-05	1.000	-2.362E-05	4.838E-05
	HC	FBTCS-Y	-1.181E-05	0.166	-2.677E-05	3.147E-06	-0.008	0.460	-0.023	0.006	-5.775E-06	0.920	-1.973E-05	8.183E-06	-2.388E-05	0.093	-5.061E-05	2.844E-06
Inferior fronto occipital (IFO)	FBTCS-N	FBTCS-Y	**2.502E-5**[*]	**0.044**	**5.022E-07**	**4.953E-05**	-0.010	0.574	-0.030	0.009	2.318E-05	0.102	-3.230E-06	4.958E-05	**2.870E-5**[*]	**0.049**	**9.479E-08**	**5.731E-05**
		HC	**3.247E-5**[*]	**0.005**	**8.291E-06**	**5.665E-05**	-0.016	0.129	-0.036	0.003	**3.344E-5**[*]	**0.008**	**7.390E-06**	**5.948E-05**	**3.055E-5**[*]	**0.030**	**2.331E-06**	**5.877E-05**
	HC	FBTCS-Y	-7.455E-06	0.914	-2.540E-05	1.049E-05	0.006	0.964	-0.009	0.020	-1.026E-05	0.575	-2.959E-05	9.073E-06	-1.846E-06	1.000	-2.279E-05	1.910E-05
ST_ Fronto orbital (STFO)	FBTCS-N	FBTCS-Y	1.630E-05	0.379	-9.818E-06	4.241E-05	-0.009	0.682	-0.028	0.010	1.623E-05	0.423	-1.080E-05	4.325E-05	1.644E-05	0.637	-1.603E-05	4.890E-05
		HC	2.542E-05	0.054	-3.393E-07	5.118E-05	-0.014	0.191	-0.033	0.004	**2.690E-5**[*]	**0.047**	**2.381E-07**	**5.355E-05**	2.247E-05	0.261	-9.547E-06	5.449E-05
	HC	FBTCS-Y	-9.123E-06	0.719	-2.824E-05	9.999E-06	0.005	1.000	-0.009	0.019	-1.067E-05	0.555	-3.045E-05	9.120E-06	-6.035E-06	1.000	-2.980E-05	1.773E-05
ST_Occipital (STOCC)	FBTCS-N	FBTCS-Y	**2.865E-5**[*]	**0.032**	**1.995E-06**	**5.531E-05**	-0.012	0.524	-0.035	0.010	2.652E-05	0.091	-3.016E-06	5.605E-05	**3.292E-5**[*]	**0.033**	**2.084E-06**	**6.376E-05**
		HC	**3.628E-5**[*]	**0.004**	**9.981E-06**	**6.257E-05**	-0.017	0.177	-0.039	0.005	**3.674E-5**[*]	**0.009**	**7.610E-06**	**6.587E-05**	**3.534E-5**[*]	**0.018**	**4.925E-06**	**6.576E-05**
	HC	FBTCS-Y	-7.622E-06	1.000	-2.714E-05	1.190E-05	0.005	1.000	-0.012	0.021	-1.022E-05	0.731	-3.185E-05	1.140E-05	-2.420E-06	1.000	-2.500E-05	2.016E-05
ST_Parietal (STPAR)	FBTCS-N	FBTCS-Y	2.146E-05	0.077	-1.703E-06	4.463E-05	-0.009	0.826	-0.030	0.012	1.962E-05	0.194	-6.207E-06	4.545E-05	2.514E-05	0.081	-2.259E-06	5.254E-05
		HC	**2.721E-5**[*]	**0.015**	**4.364E-06**	**5.007E-05**	-0.017	0.125	-0.038	0.003	**2.941E-5**[*]	**0.019**	**3.933E-06**	**5.489E-05**	2.282E-05	0.123	-4.207E-06	4.984E-05
	HC	FBTCS-Y	-5.752E-06	1.000	-2.271E-05	1.121E-05	0.008	0.578	-0.007	0.023	-9.787E-06	0.608	-2.870E-05	9.124E-06	2.322E-06	1.000	-1.774E-05	2.238E-05
ST_Postcentral (STPOSTC)	FBTCS-N	FBTCS-Y	1.122E-05	0.644	-1.105E-05	3.348E-05	-0.007	1.000	-0.028	0.015	1.006E-05	0.932	-1.446E-05	3.458E-05	1.353E-05	0.846	-1.752E-05	4.458E-05
		HC	2.154E-05	0.056	-4.222E-07	4.350E-05	-0.015	0.293	-0.036	0.007	2.271E-05	0.072	-1.470E-06	4.690E-05	1.919E-05	0.374	-1.143E-05	4.982E-05
	HC	FBTCS-Y	-1.032E-05	0.363	-2.663E-05	5.978E-06	0.008	0.650	-0.008	0.024	-1.266E-05	0.256	-3.061E-05	5.295E-06	-5.662E-06	1.000	-2.839E-05	1.707E-05
ST_Precentral (STPREC)	FBTCS-N	FBTCS-Y	1.400E-05	0.422	-9.298E-06	3.729E-05	-0.009	0.887	-0.029	0.012	1.368E-05	0.548	-1.156E-05	3.892E-05	1.463E-05	0.704	-1.572E-05	4.498E-05
		HC	**2.571E-5**[*]	**0.024**	**2.731E-06**	**4.869E-05**	-0.017	0.132	-0.037	0.003	**2.753E-5**[*]	**0.026**	**2.635E-06**	**5.243E-05**	2.206E-05	0.218	-7.881E-06	5.199E-05
	HC	FBTCS-Y	-1.171E-05	0.281	-2.877E-05	5.344E-06	0.008	0.548	-0.007	0.023	-1.385E-05	0.204	-3.234E-05	4.626E-06	-7.422E-06	1.000	-2.964E-05	1.480E-05
ST_Prefrontal (STPREF)	FBTCS-N	FBTCS-Y	1.795E-05	0.219	-6.418E-06	4.231E-05	-0.010	0.493	-0.027	0.007	1.792E-05	0.245	-7.188E-06	4.304E-05	1.799E-05	0.394	-1.124E-05	4.723E-05
		HC	**2.785E-5**[*]	**0.018**	**3.819E-06**	**5.189E-05**	-0.016	0.075	-0.032	0.001	**2.987E-5**[*]	**0.013**	**5.099E-06**	**5.464E-05**	2.381E-05	0.136	-5.021E-06	5.265E-05
	HC	FBTCS-Y	-9.905E-06	0.517	-2.774E-05	7.934E-06	0.006	0.705	-0.006	0.018	-1.195E-05	0.336	-3.033E-05	6.441E-06	-5.820E-06	1.000	-2.722E-05	1.558E-05
ST_Premotor (STPREM)	FBTCS-N	FBTCS-Y	1.679E-05	0.294	-7.986E-06	4.156E-05	-0.011	0.374	-0.028	0.006	1.762E-05	0.260	-7.480E-06	4.272E-05	1.512E-05	0.670	-1.548E-05	4.572E-05
		HC	**2.728E-5**[*]	**0.024**	**2.848E-06**	**5.172E-05**	-0.016	0.068	-0.033	0.001	**2.911E-5**[*]	**0.016**	**4.358E-06**	**5.387E-05**	2.362E-05	0.172	-6.560E-06	5.380E-05
	HC	FBTCS-Y	-1.050E-05	0.466	-2.863E-05	7.641E-06	0.005	0.895	-0.007	0.018	-1.150E-05	0.376	-2.987E-05	6.880E-06	-8.503E-06	1.000	-3.091E-05	1.390E-05
T_Occipital (TOCC)	FBTCS-N	FBTCS-Y	**2.842E-5**[*]	**0.042**	**7.677E-07**	**5.607E-05**	-0.013	0.400	-0.034	0.008	2.715E-05	0.088	-2.878E-06	5.718E-05	3.097E-05	0.061	-1.027E-06	6.296E-05
		HC	**3.822E-5**[*]	**0.004**	**1.095E-05**	**6.550E-05**	-0.019	0.089	-0.040	0.002	**3.993E-5**[*]	**0.005**	**1.031E-05**	**6.955E-05**	**3.481E-5**[*]	**0.026**	**3.255E-06**	**6.637E-05**
	HC	FBTCS-Y	-9.803E-06	0.698	-3.005E-05	1.044E-05	0.006	1.000	-0.010	0.021	-1.278E-05	0.461	-3.477E-05	9.204E-06	-3.846E-06	1.000	-2.727E-05	1.958E-05
T_Postcentral (TPOSTC)	FBTCS-N	FBTCS-Y	1.158E-05	0.515	-9.240E-06	3.241E-05	-0.008	1.000	-0.031	0.014	1.145E-05	0.691	-1.208E-05	3.498E-05	1.185E-05	1.000	-1.956E-05	4.326E-05
		HC	**2.198E-5**[*]	**0.032**	**1.445E-06**	**4.252E-05**	-0.016	0.263	-0.038	0.007	**2.376E-5**[*]	**0.043**	**5.505E-07**	**4.696E-05**	1.844E-05	0.433	-1.254E-05	4.943E-05
	HC	FBTCS-Y	-1.040E-05	0.287	-2.565E-05	4.845E-06	0.007	0.843	-0.009	0.024	-1.231E-05	0.245	-2.953E-05	4.918E-06	-6.594E-06	1.000	-2.959E-05	1.640E-05
T_Precentral (TPREC)	FBTCS-N	FBTCS-Y	1.390E-05	0.396	-8.714E-06	3.650E-05	-0.010	0.727	-0.031	0.011	1.448E-05	0.459	-1.040E-05	3.937E-05	1.272E-05	0.913	-1.787E-05	4.331E-05
		HC	**2.650E-5**[*]	**0.015**	**4.195E-06**	**4.880E-05**	-0.018	0.098	-0.039	0.002	**2.927E-5**[*]	**0.015**	**4.731E-06**	**5.381E-05**	2.094E-05	0.271	-9.231E-06	5.111E-05
	HC	FBTCS-Y	-1.260E-05	0.193	-2.915E-05	3.953E-06	0.008	0.541	-0.007	0.024	-1.479E-05	0.147	-3.301E-05	3.429E-06	-8.222E-06	1.000	-3.062E-05	1.417E-05
T_Prefrontal (TPREF)	FBTCS-N	FBTCS-Y	1.839E-05	0.187	-5.582E-06	4.235E-05	-0.011	0.349	-0.028	0.006	1.908E-05	0.177	-5.476E-06	4.363E-05	1.699E-05	0.475	-1.259E-05	4.656E-05
		HC	**2.958E-5**[*]	**0.010**	**5.941E-06**	**5.322E-05**	**-.017**[*]	**0.048**	**-0.033**	**0.000**	**3.217E-5**[*]	**0.006**	**7.951E-06**	**5.639E-05**	2.440E-05	0.129	-4.774E-06	5.357E-05
	HC	FBTCS-Y	-1.120E-05	0.355	-2.874E-05	6.352E-06	0.006	0.715	-0.006	0.018	-1.309E-05	0.228	-3.107E-05	4.886E-06	-7.411E-06	1.000	-2.907E-05	1.424E-05
T_Premotor (TPREM)	FBTCS-N	FBTCS-Y	1.729E-05	0.212	-5.994E-06	4.057E-05	-0.014	0.157	-0.030	0.003	1.981E-05	0.121	-3.557E-06	4.318E-05	1.226E-05	0.969	-1.840E-05	4.291E-05
		HC	**2.917E-5**[*]	**0.009**	**6.206E-06**	**5.214E-05**	**-.018**[*]	**0.031**	**-0.035**	**-0.001**	**3.215E-5**[*]	**0.004**	**9.106E-06**	**5.520E-05**	2.322E-05	0.186	-7.018E-06	5.345E-05
	HC	FBTCS-Y	-1.188E-05	0.267	-2.893E-05	5.165E-06	0.004	1.000	-0.008	0.017	-1.234E-05	0.236	-2.945E-05	4.764E-06	-1.096E-05	0.686	-3.341E-05	1.148E-05
Uncinate fascicle (UF)	FBTCS-N	FBTCS-Y	1.687E-05	0.341	-9.223E-06	4.296E-05	-0.001	1.000	-0.020	0.018	1.159E-05	0.826	-1.463E-05	3.780E-05	2.739E-05	0.163	-7.156E-06	6.193E-05
		HC	2.542E-05	0.054	-3.147E-07	5.116E-05	-0.012	0.338	-0.031	0.007	2.508E-05	0.060	-7.819E-07	5.094E-05	2.607E-05	0.189	-7.999E-06	6.014E-05
	HC	FBTCS-Y	-8.553E-06	0.808	-2.766E-05	1.055E-05	0.012	0.133	-0.002	0.025	-1.349E-05	0.259	-3.269E-05	5.703E-06	1.314E-06	1.000	-2.398E-05	2.660E-05
Commissure anterior (CA)	FBTCS-N	FBTCS-Y	1.305E-05	0.672	-1.340E-05	3.951E-05	-0.014	0.508	-0.040	0.011	1.632E-05	0.553	-1.390E-05	4.654E-05	6.524E-06	1.000	-3.029E-05	4.334E-05
		HC	1.884E-05	0.236	-7.257E-06	4.494E-05	-0.014	0.561	-0.039	0.012	2.053E-05	0.278	-9.275E-06	5.034E-05	1.545E-05	0.880	-2.086E-05	5.176E-05
	HC	FBTCS-Y	-5.786E-06	1.000	-2.516E-05	1.359E-05	-0.001	1.000	-0.020	0.018	-4.215E-06	1.000	-2.634E-05	1.791E-05	-8.926E-06	1.000	-3.588E-05	1.803E-05
Corpus callosum (CC)	FBTCS-N	FBTCS-Y	2.202E-05	0.075	-1.600E-06	4.563E-05	-0.011	0.387	-0.030	0.007	2.208E-05	0.110	-3.463E-06	4.762E-05	2.189E-05	0.133	-4.479E-06	4.827E-05
		HC	**2.852E-5**[*]	**0.012**	**5.221E-06**	**5.181E-05**	**-.019**[*]	**0.043**	**-0.037**	**0.000**	**3.203E-5**[*]	**0.009**	**6.837E-06**	**5.723E-05**	2.148E-05	0.136	-4.528E-06	4.750E-05
	HC	FBTCS-Y	-6.499E-06	1.000	-2.379E-05	1.079E-05	0.007	0.564	-0.006	0.021	-9.952E-06	0.572	-2.865E-05	8.750E-06	4.087E-07	1.000	-1.890E-05	1.972E-05

Key: HC: control group; Mean difference: the difference between group 1 and group 2, based on the estimated marginal means of the univariate ANCOVA (Bonferroni correction selected). Positive mean difference values indicate group 1 had a higher average TW-TM than group 2, negative values indicate group 2 had a higher average TW-TM than group 1. "*p*" represents the significance of the mean difference.*a significant difference was observed in the estimated marginal means univariate ANCOVA output.

Supplementary Table 4. Group differences between the mean TW-TM of each left and right tract in the FBTCS-N, FBTCS-Y and control groups.

			TW-ADC							
			LEFT				RIGHT			
Tract	Group 1	Group 2	Mean Difference	*p*	95% CI Lower	95% CI Upper	Mean Difference	*p*	95% CI Lower	95% CI Upper
Anterior thalamic radiation (ATR)	FBTCS-N	FBTCS-Y	1.696E-05	0.258	-7.150E-06	4.107E-05	1.842E-05	0.203	-6.094E-06	4.294E-05
		HC	**2.713E-5***	**0.021**	**3.354E-06**	**5.091E-05**	**3.119E-5***	**0.008**	**7.009E-06**	**5.537E-05**
	HC	FBTCS-Y	-1.017E-05	0.471	-2.783E-05	7.476E-06	-1.277E-05	0.248	-3.072E-05	5.181E-06
Superior thalamic radiation (STR)	FBTCS-N	FBTCS-Y	1.201E-05	0.664	-1.218E-05	3.619E-05	8.251E-06	1.000	-1.726E-05	3.376E-05
		HC	**2.566E-5***	**0.031**	**1.804E-06**	**4.951E-05**	2.282E-05	0.087	-2.347E-06	4.798E-05
	HC	FBTCS-Y	-1.365E-05	0.183	-3.136E-05	4.054E-06	-1.457E-05	0.175	-3.325E-05	4.113E-06
Corticospinal (CST)	FBTCS-N	FBTCS-Y	1.505E-05	0.197	-4.841E-06	3.494E-05	1.177E-05	0.490	-8.969E-06	3.251E-05
		HC	**2.138E-5***	**0.029**	**1.758E-06**	**4.100E-05**	1.919E-05	0.072	-1.267E-06	3.965E-05
	HC	FBTCS-Y	-6.328E-06	0.850	-2.089E-05	8.235E-06	-7.419E-06	0.686	-2.260E-05	7.766E-06
Fronto pontine (FPT)	FBTCS-N	FBTCS-Y	1.654E-05	0.193	-5.206E-06	3.830E-05	1.720E-05	0.156	-4.288E-06	3.869E-05
		HC	**2.309E-5***	**0.031**	**1.635E-06**	**4.454E-05**	**2.590E-5***	**0.012**	**4.706E-06**	**4.710E-05**
	HC	FBTCS-Y	-6.545E-06	0.930	-2.247E-05	9.380E-06	-8.701E-06	0.522	-2.443E-05	7.033E-06
Parieto-occipital pontine (POPT)	FBTCS-N	FBTCS-Y	1.724E-05	0.127	-3.321E-06	3.779E-05	1.705E-05	0.191	-5.319E-06	3.943E-05
		HC	**2.293E-5***	**0.022**	**2.657E-06**	**4.321E-05**	**2.230E-5***	**0.047**	**2.322E-07**	**4.437E-05**
	HC	FBTCS-Y	-5.697E-06	1.000	-2.075E-05	9.353E-06	-5.246E-06	1.000	-2.163E-05	1.113E-05
Inferior cerebellar peduncle (ICP)	FBTCS-N	FBTCS-Y	5.931E-06	1.000	-1.340E-05	2.526E-05	9.522E-06	0.893	-1.306E-05	3.210E-05
		HC	1.048E-05	0.530	-8.584E-06	2.955E-05	1.678E-05	0.201	-5.498E-06	3.905E-05
	HC	FBTCS-Y	-4.551E-06	1.000	-1.870E-05	9.601E-06	-7.253E-06	0.836	-2.379E-05	9.280E-06
Superior cerebellar peduncle (SCP)	FBTCS-N	FBTCS-Y	2.142E-06	1.000	-1.836E-05	2.265E-05	5.616E-06	1.000	-1.577E-05	2.700E-05
		HC	1.453E-05	0.240	-5.698E-06	3.475E-05	1.697E-05	0.153	-4.116E-06	3.806E-05
	HC	FBTCS-Y	-1.239E-05	0.137	-2.740E-05	2.627E-06	-1.136E-05	0.231	-2.701E-05	4.297E-06
Inferior fronto occipital (IFO)	FBTCS-N	FBTCS-Y	2.376E-05	0.073	-1.636E-06	4.915E-05	**2.606E-5***	**0.047**	**2.252E-07**	**5.190E-05**
		HC	**3.054E-5***	**0.012**	**5.497E-06**	**5.559E-05**	**3.490E-5***	**0.004**	**9.410E-06**	**6.038E-05**
	HC	FBTCS-Y	-6.787E-06	1.000	-2.538E-05	1.181E-05	-8.833E-06	0.748	-2.775E-05	1.008E-05
ST_ Fronto orbital (STFO)	FBTCS-N	FBTCS-Y	1.286E-05	0.682	-1.338E-05	3.910E-05	2.059E-05	0.200	-6.720E-06	4.789E-05
		HC	2.167E-05	0.128	-4.220E-06	4.755E-05	**3.031E-5***	**0.023**	**3.380E-06**	**5.725E-05**
	HC	FBTCS-Y	-8.805E-06	0.775	-2.802E-05	1.041E-05	-9.727E-06	0.692	-2.972E-05	1.027E-05
ST_Occipital (STOCC)	FBTCS-N	FBTCS-Y	2.958E-05	0.055	-4.475E-07	5.962E-05	2.816E-05	0.059	-8.203E-07	5.713E-05
		HC	**3.509E-5***	**0.016**	**5.470E-06**	**6.471E-05**	**3.962E-5***	**0.004**	**1.104E-05**	**6.820E-05**
	HC	FBTCS-Y	-5.508E-06	1.000	-2.750E-05	1.648E-05	-1.147E-05	0.551	-3.268E-05	9.748E-06
ST_Parietal (STPAR)	FBTCS-N	FBTCS-Y	2.019E-05	0.117	-3.473E-06	4.385E-05	2.206E-05	0.099	-2.910E-06	4.702E-05
		HC	**2.678E-5***	**0.020**	**3.442E-06**	**5.012E-05**	**2.770E-5***	**0.023**	**3.074E-06**	**5.233E-05**
	HC	FBTCS-Y	-6.592E-06	1.000	-2.392E-05	1.073E-05	-5.643E-06	1.000	-2.392E-05	1.264E-05
ST_Postcentral (STPOSTC)	FBTCS-N	FBTCS-Y	1.100E-05	0.641	-1.077E-05	3.276E-05	1.104E-05	0.815	-1.375E-05	3.583E-05
		HC	**2.282E-5***	**0.034**	**1.345E-06**	**4.429E-05**	1.987E-05	0.146	-4.576E-06	4.433E-05
	HC	FBTCS-Y	-1.182E-05	0.213	-2.776E-05	4.117E-06	-8.837E-06	0.691	-2.699E-05	9.313E-06
ST_Precentral (STPREC)	FBTCS-N	FBTCS-Y	1.424E-05	0.415	-9.328E-06	3.781E-05	1.332E-05	0.563	-1.155E-05	3.820E-05
		HC	**2.668E-5***	**0.020**	**3.436E-06**	**4.993E-05**	2.441E-05	0.052	-1.292E-07	4.894E-05
	HC	FBTCS-Y	-1.244E-05	0.237	-2.970E-05	4.814E-06	-1.108E-05	0.407	-2.929E-05	7.130E-06
ST_Prefrontal (STPREF)	FBTCS-N	FBTCS-Y	1.657E-05	0.287	-7.722E-06	4.085E-05	1.923E-05	0.183	-5.706E-06	4.416E-05
		HC	**2.571E-5***	**0.032**	**1.751E-06**	**4.966E-05**	**3.005E-5***	**0.012**	**5.457E-06**	**5.465E-05**
	HC	FBTCS-Y	-9.142E-06	0.617	-2.692E-05	8.641E-06	-1.082E-05	0.437	-2.908E-05	7.433E-06
ST_Premotor (STPREM)	FBTCS-N	FBTCS-Y	1.707E-05	0.298	-8.243E-06	4.238E-05	1.608E-05	0.356	-9.144E-06	4.130E-05
		HC	**2.708E-5***	**0.030**	**2.118E-06**	**5.205E-05**	**2.734E-5***	**0.027**	**2.466E-06**	**5.222E-05**
	HC	FBTCS-Y	-1.002E-05	0.552	-2.855E-05	8.516E-06	-1.127E-05	0.404	-2.973E-05	7.201E-06
T_Occipital (TOCC)	FBTCS-N	FBTCS-Y	3.116E-05	0.066	-1.527E-06	6.384E-05	2.652E-05	0.106	-3.925E-06	5.697E-05
		HC	**3.818E-5***	**0.016**	**5.947E-06**	**7.042E-05**	**4.145E-5***	**0.004**	**1.142E-05**	**7.148E-05**
	HC	FBTCS-Y	-7.029E-06	1.000	-3.096E-05	1.690E-05	-1.493E-05	0.305	-3.722E-05	7.359E-06
T_Postcentral (TPOSTC)	FBTCS-N	FBTCS-Y	1.201E-05	0.445	-8.387E-06	3.241E-05	1.065E-05	0.794	-1.292E-05	3.423E-05
		HC	**2.353E-5***	**0.017**	**3.412E-06**	**4.365E-05**	2.020E-05	0.108	-3.052E-06	4.345E-05
	HC	FBTCS-Y	-1.152E-05	0.182	-2.646E-05	3.414E-06	-9.547E-06	0.522	-2.681E-05	7.712E-06
T_Precentral (TPREC)	FBTCS-N	FBTCS-Y	1.514E-05	0.312	-7.617E-06	3.789E-05	1.223E-05	0.641	-1.198E-05	3.643E-05
		HC	**2.749E-5***	**0.012**	**5.047E-06**	**4.993E-05**	**2.532E-5***	**0.034**	**1.448E-06**	**4.920E-05**
	HC	FBTCS-Y	-1.235E-05	0.213	-2.901E-05	4.305E-06	-1.310E-05	0.216	-3.082E-05	4.626E-06
T_Prefrontal (TPREF)	FBTCS-N	FBTCS-Y	1.778E-05	0.214	-6.209E-06	4.177E-05	1.883E-05	0.187	-5.724E-06	4.338E-05
		HC	**2.797E-5***	**0.016**	**4.304E-06**	**5.163E-05**	**3.123E-5***	**0.008**	**7.013E-06**	**5.545E-05**
	HC	FBTCS-Y	-1.019E-05	0.464	-2.775E-05	7.377E-06	-1.240E-05	0.276	-3.038E-05	5.575E-06
T_Premotor (TPREM)	FBTCS-N	FBTCS-Y	1.761E-05	0.230	-6.626E-06	4.184E-05	1.659E-05	0.250	-6.774E-06	3.995E-05
		HC	**2.833E-5***	**0.015**	**4.424E-06**	**5.223E-05**	**3.008E-5***	**0.007**	**7.035E-06**	**5.313E-05**
	HC	FBTCS-Y	-1.072E-05	0.415	-2.846E-05	7.023E-06	-1.349E-05	0.167	-3.060E-05	3.616E-06
Uncinate fascicle (UF)	FBTCS-N	FBTCS-Y	1.475E-05	0.445	-1.028E-05	3.979E-05	1.884E-05	0.316	-9.592E-06	4.728E-05
		HC	2.441E-05	0.054	-2.865E-07	4.911E-05	2.755E-05	0.056	-4.956E-07	5.560E-05
	HC	FBTCS-Y	-9.656E-06	0.585	-2.799E-05	8.676E-06	-8.708E-06	0.904	-2.953E-05	1.211E-05

			TW-FA							
			LEFT				RIGHT			
Tract	Group 1	Group 2	Mean Difference	*p*	95% CI Lower	95% CI Upper	Mean Difference	*p*	95% CI Lower	95% CI Upper
Anterior thalamic radiation (ATR)	FBTCS-N	FBTCS-Y	-0.012	0.247	-0.030	0.005	-0.008	0.729	-0.024	0.009
		HC	-0.015	0.098	-0.032	0.002	-0.016	0.054	-0.032	0.000
	HC	FBTCS-Y	0.003	1.000	-0.010	0.015	0.008	0.285	-0.004	0.020
Superior thalamic radiation (STR)	FBTCS-N	FBTCS-Y	-0.007	1.000	-0.033	0.018	-0.015	0.413	-0.039	0.010
		HC	-0.019	0.215	-0.044	0.007	-0.020	0.138	-0.044	0.004
	HC	FBTCS-Y	0.011	0.414	-0.007	0.030	0.005	1.000	-0.013	0.023
Corticospinal (CST)	FBTCS-N	FBTCS-Y	-0.008	1.000	-0.029	0.013	-0.009	0.793	-0.028	0.011
		HC	-0.014	0.295	-0.034	0.007	-0.014	0.203	-0.034	0.005
	HC	FBTCS-Y	0.006	0.986	-0.009	0.021	0.006	0.991	-0.009	0.020
Fronto pontine (FPT)	FBTCS-N	FBTCS-Y	-0.009	0.570	-0.026	0.008	-0.012	0.220	-0.028	0.004
		HC	-0.014	0.146	-0.030	0.003	**-.016**[*]	**0.042**	**-0.032**	**0.000**
	HC	FBTCS-Y	0.005	1.000	-0.008	0.017	0.004	1.000	-0.007	0.016
Parieto-occipital pontine (POPT)	FBTCS-N	FBTCS-Y	-0.008	0.977	-0.028	0.012	-0.010	0.641	-0.029	0.009
		HC	-0.016	0.141	-0.036	0.004	-0.014	0.202	-0.033	0.005
	HC	FBTCS-Y	0.008	0.502	-0.006	0.023	0.004	1.000	-0.009	0.018
Inferior cerebellar peduncle (ICP)	FBTCS-N	FBTCS-Y	-0.019	0.168	-0.043	0.005	-0.016	0.210	-0.037	0.005
		HC	-0.010	0.854	-0.034	0.013	-0.009	0.819	-0.030	0.012
	HC	FBTCS-Y	-0.009	0.680	-0.026	0.009	-0.006	0.905	-0.022	0.009
Superior cerebellar peduncle (SCP)	FBTCS-N	FBTCS-Y	-0.019	0.108	-0.040	0.003	**-.019**[*]	**0.048**	**-0.038**	**0.000**
		HC	-0.010	0.780	-0.031	0.012	-0.011	0.439	-0.030	0.008
	HC	FBTCS-Y	-0.009	0.483	-0.025	0.007	-0.008	0.466	-0.022	0.006
Inferior fronto occipital (IFO)	FBTCS-N	FBTCS-Y	-0.012	0.455	-0.033	0.009	-0.008	0.878	-0.028	0.011
		HC	-0.017	0.122	-0.037	0.003	-0.015	0.181	-0.035	0.004
	HC	FBTCS-Y	0.005	1.000	-0.010	0.020	0.007	0.770	-0.008	0.021
ST_ Fronto orbital (STFO)	FBTCS-N	FBTCS-Y	-0.011	0.530	-0.031	0.009	-0.007	1.000	-0.026	0.012
		HC	-0.014	0.263	-0.033	0.006	-0.015	0.147	-0.033	0.003
	HC	FBTCS-Y	0.003	1.000	-0.012	0.017	0.008	0.473	-0.006	0.022
ST_Occipital (STOCC)	FBTCS-N	FBTCS-Y	-0.010	0.908	-0.034	0.014	-0.013	0.450	-0.036	0.009
		HC	-0.018	0.203	-0.042	0.006	-0.015	0.290	-0.038	0.007
	HC	FBTCS-Y	0.008	0.827	-0.010	0.026	0.002	1.000	-0.015	0.019
ST_Parietal (STPAR)	FBTCS-N	FBTCS-Y	-0.009	0.904	-0.030	0.012	-0.010	0.786	-0.031	0.012
		HC	-0.018	0.099	-0.039	0.002	-0.017	0.173	-0.038	0.005
	HC	FBTCS-Y	0.009	0.390	-0.006	0.025	0.007	0.859	-0.009	0.022
ST_Postcentral (STPOSTC)	FBTCS-N	FBTCS-Y	-0.007	1.000	-0.028	0.015	-0.006	1.000	-0.029	0.017
		HC	-0.017	0.168	-0.038	0.005	-0.012	0.534	-0.035	0.010
	HC	FBTCS-Y	0.010	0.377	-0.006	0.026	0.006	1.000	-0.011	0.023
ST_Precentral (STPREC)	FBTCS-N	FBTCS-Y	-0.008	0.961	-0.029	0.012	-0.009	0.886	-0.030	0.012
		HC	-0.018	0.110	-0.038	0.003	-0.016	0.201	-0.037	0.005
	HC	FBTCS-Y	0.009	0.395	-0.006	0.024	0.007	0.844	-0.009	0.022
ST_Prefrontal (STPREF)	FBTCS-N	FBTCS-Y	-0.010	0.502	-0.027	0.008	-0.009	0.526	-0.026	0.008
		HC	-0.015	0.107	-0.032	0.002	-0.016	0.058	-0.033	0.000
	HC	FBTCS-Y	0.005	0.952	-0.008	0.018	0.007	0.503	-0.005	0.019
ST_Premotor (STPREM)	FBTCS-N	FBTCS-Y	-0.011	0.348	-0.029	0.006	-0.010	0.490	-0.028	0.008
		HC	-0.016	0.083	-0.034	0.001	-0.017	0.075	-0.034	0.001
	HC	FBTCS-Y	0.005	1.000	-0.008	0.018	0.006	0.712	-0.007	0.020
T_Occipital (TOCC)	FBTCS-N	FBTCS-Y	-0.011	0.768	-0.033	0.012	-0.015	0.286	-0.037	0.007
		HC	-0.020	0.096	-0.043	0.002	-0.017	0.165	-0.039	0.005
	HC	FBTCS-Y	0.010	0.487	-0.007	0.026	0.002	1.000	-0.014	0.018
T_Postcentral (TPOSTC)	FBTCS-N	FBTCS-Y	-0.008	1.000	-0.030	0.014	-0.009	1.000	-0.032	0.015
		HC	-0.018	0.130	-0.040	0.004	-0.013	0.537	-0.036	0.011
	HC	FBTCS-Y	0.010	0.395	-0.006	0.026	0.004	1.000	-0.013	0.021
T_Precentral (TPREC)	FBTCS-N	FBTCS-Y	-0.009	0.907	-0.030	0.012	-0.011	0.609	-0.033	0.010
		HC	-0.019	0.093	-0.040	0.002	-0.018	0.127	-0.039	0.003
	HC	FBTCS-Y	0.010	0.361	-0.006	0.026	0.007	0.890	-0.009	0.022
T_Prefrontal (TPREF)	FBTCS-N	FBTCS-Y	-0.011	0.372	-0.028	0.006	-0.011	0.365	-0.027	0.006
		HC	-0.016	0.067	-0.033	0.001	**-.017**[*]	**0.040**	**-0.034**	**-0.001**
	HC	FBTCS-Y	0.005	0.883	-0.007	0.018	0.007	0.570	-0.006	0.019
T_Premotor (TPREM)	FBTCS-N	FBTCS-Y	-0.013	0.187	-0.030	0.004	-0.014	0.157	-0.031	0.004
		HC	-0.017	0.052	-0.034	0.000	**-.019**[*]	**0.024**	**-0.036**	**-0.002**
	HC	FBTCS-Y	0.004	1.000	-0.009	0.016	0.005	0.915	-0.007	0.018
Uncinate fascicle (UF)	FBTCS-N	FBTCS-Y	-0.005	1.000	-0.026	0.015	0.004	1.000	-0.015	0.023
		HC	-0.014	0.267	-0.034	0.006	-0.011	0.462	-0.030	0.008
	HC	FBTCS-Y	0.009	0.476	-0.006	0.024	**.015**[*]	**0.030**	**0.001**	**0.029**

			TW-RD							
			LEFT				RIGHT			
Tract	Group 1	Group 2	Mean Difference	*p*	95% CI Lower	95% CI Upper	Mean Difference	*p*	95% CI Lower	95% CI Upper
Anterior thalamic radiation (ATR)	FBTCS-N	FBTCS-Y	1.883E-05	0.165	-4.989E-06	4.264E-05	1.763E-05	0.240	-6.929E-06	4.218E-05
		HC	**2.946E-5[*]**	**0.010**	**5.967E-06**	**5.295E-05**	**3.363E-5[*]**	**0.004**	**9.414E-06**	**5.786E-05**
	HC	FBTCS-Y	-1.063E-05	0.405	-2.807E-05	6.806E-06	-1.601E-05	0.095	-3.399E-05	1.971E-06
Corticospinal (CST)	FBTCS-N	FBTCS-Y	1.393E-05	0.413	-9.074E-06	3.694E-05	1.199E-05	0.571	-1.052E-05	3.451E-05
		HC	**2.272E-5[*]**	**0.050**	**2.566E-08**	**4.541E-05**	2.129E-05	0.064	-9.221E-07	4.349E-05
	HC	FBTCS-Y	-8.786E-06	0.598	-2.563E-05	8.057E-06	-9.295E-06	0.498	-2.578E-05	7.190E-06
Fronto pontine (FPT)	FBTCS-N	FBTCS-Y	1.612E-05	0.261	-6.870E-06	3.911E-05	1.846E-05	0.142	-4.100E-06	4.102E-05
		HC	**2.471E-5[*]**	**0.029**	**2.031E-06**	**4.739E-05**	**2.884E-5[*]**	**0.007**	**6.586E-06**	**5.109E-05**
	HC	FBTCS-Y	-8.587E-06	0.627	-2.542E-05	8.247E-06	-1.038E-05	0.371	-2.689E-05	6.138E-06
Inferior cerebellar peduncle (ICP)	FBTCS-N	FBTCS-Y	1.278E-05	0.526	-1.039E-05	3.594E-05	1.448E-05	0.410	-9.363E-06	3.832E-05
		HC	1.285E-05	0.501	-9.999E-06	3.570E-05	1.802E-05	0.188	-5.498E-06	4.154E-05
	HC	FBTCS-Y	-7.573E-08	1.000	-1.704E-05	1.689E-05	-3.540E-06	1.000	-2.100E-05	1.392E-05
Inferior fronto occipital (IFO)	FBTCS-N	FBTCS-Y	2.319E-05	0.121	-4.159E-06	5.054E-05	2.280E-05	0.135	-4.742E-06	5.034E-05
		HC	**3.251E-5[*]**	**0.014**	**5.534E-06**	**5.949E-05**	**3.470E-5[*]**	**0.008**	**7.537E-06**	**6.186E-05**
	HC	FBTCS-Y	-9.320E-06	0.753	-2.934E-05	1.070E-05	-1.190E-05	0.442	-3.207E-05	8.259E-06
Parieto-occipital pontine (POPT)	FBTCS-N	FBTCS-Y	1.581E-05	0.290	-7.432E-06	3.904E-05	1.596E-05	0.289	-7.492E-06	3.941E-05
		HC	**2.565E-5[*]**	**0.024**	**2.726E-06**	**4.857E-05**	**2.349E-5[*]**	**0.045**	**3.589E-07**	**4.662E-05**
	HC	FBTCS-Y	-9.841E-06	0.467	-2.685E-05	7.173E-06	-7.531E-06	0.836	-2.470E-05	9.637E-06
Superior cerebellar peduncle (SCP)	FBTCS-N	FBTCS-Y	9.832E-06	0.648	-9.746E-06	2.941E-05	1.364E-05	0.276	-6.125E-06	3.341E-05
		HC	1.578E-05	0.143	-3.526E-06	3.510E-05	1.925E-05	0.054	-2.442E-07	3.875E-05
	HC	FBTCS-Y	-5.953E-06	0.914	-2.029E-05	8.381E-06	-5.611E-06	1.000	-2.008E-05	8.860E-06
ST_ Fronto orbital (STFO)	FBTCS-N	FBTCS-Y	1.421E-05	0.587	-1.281E-05	4.123E-05	1.866E-05	0.313	-9.416E-06	4.673E-05
		HC	2.338E-05	0.102	-3.269E-06	5.003E-05	**3.140E-5[*]**	**0.022**	**3.706E-06**	**5.908E-05**
	HC	FBTCS-Y	-9.173E-06	0.758	-2.896E-05	1.061E-05	-1.274E-05	0.387	-3.329E-05	7.815E-06
ST_Occipital (STOCC)	FBTCS-N	FBTCS-Y	2.617E-05	0.143	-5.875E-06	5.821E-05	2.648E-05	0.114	-4.387E-06	5.734E-05
		HC	**3.632E-5[*]**	**0.020**	**4.715E-06**	**6.793E-05**	**3.813E-5[*]**	**0.010**	**7.687E-06**	**6.858E-05**
	HC	FBTCS-Y	-1.015E-05	0.856	-3.361E-05	1.331E-05	-1.165E-05	0.613	-3.425E-05	1.095E-05
ST_Parietal (STPAR)	FBTCS-N	FBTCS-Y	1.879E-05	0.240	-7.358E-06	4.493E-05	2.000E-05	0.220	-7.199E-06	4.719E-05
		HC	**2.978E-5[*]**	**0.019**	**3.993E-06**	**5.557E-05**	**2.910E-5[*]**	**0.030**	**2.279E-06**	**5.593E-05**
	HC	FBTCS-Y	-1.099E-05	0.475	-3.014E-05	8.148E-06	-9.107E-06	0.777	-2.902E-05	1.081E-05
ST_Postcentral (STPOSTC)	FBTCS-N	FBTCS-Y	1.020E-05	0.876	-1.370E-05	3.409E-05	9.636E-06	1.000	-1.722E-05	3.649E-05
		HC	**2.489E-5[*]**	**0.035**	**1.322E-06**	**4.845E-05**	2.014E-05	0.193	-6.344E-06	4.663E-05
	HC	FBTCS-Y	-1.469E-05	0.126	-3.218E-05	2.800E-06	-1.051E-05	0.566	-3.017E-05	9.153E-06
ST_Precentral (STPREC)	FBTCS-N	FBTCS-Y	1.361E-05	0.572	-1.197E-05	3.920E-05	1.344E-05	0.648	-1.332E-05	4.020E-05
		HC	**2.858E-5[*]**	**0.022**	**3.350E-06**	**5.382E-05**	2.617E-05	0.053	-2.239E-07	5.257E-05
	HC	FBTCS-Y	-1.497E-05	0.157	-3.370E-05	3.760E-06	-1.273E-05	0.336	-3.232E-05	6.860E-06
ST_Prefrontal (STPREF)	FBTCS-N	FBTCS-Y	1.677E-05	0.306	-8.289E-06	4.182E-05	1.895E-05	0.216	-6.681E-06	4.458E-05
		HC	**2.750E-5[*]**	**0.025**	**2.785E-06**	**5.221E-05**	**3.230E-5[*]**	**0.008**	**7.019E-06**	**5.758E-05**
	HC	FBTCS-Y	-1.073E-05	0.453	-2.908E-05	7.612E-06	-1.335E-05	0.248	-3.212E-05	5.416E-06
ST_Premotor (STPREM)	FBTCS-N	FBTCS-Y	1.786E-05	0.266	-7.735E-06	4.346E-05	1.704E-05	0.318	-8.735E-06	4.282E-05
		HC	**2.823E-5[*]**	**0.024**	**2.984E-06**	**5.348E-05**	**3.006E-5[*]**	**0.016**	**4.635E-06**	**5.549E-05**
	HC	FBTCS-Y	-1.037E-05	0.522	-2.911E-05	8.370E-06	-1.302E-05	0.277	-3.189E-05	5.855E-06
Superior thalamic radiation (STR)	FBTCS-N	FBTCS-Y	1.063E-05	1.000	-1.658E-05	3.784E-05	1.256E-05	0.767	-1.469E-05	3.982E-05
		HC	**2.855E-5[*]**	**0.034**	**1.715E-06**	**5.539E-05**	**2.770E-5[*]**	**0.041**	**8.216E-07**	**5.459E-05**
	HC	FBTCS-Y	-1.792E-05	0.090	-3.784E-05	2.001E-06	-1.514E-05	0.195	-3.510E-05	4.811E-06
T_Occipital (TOCC)	FBTCS-N	FBTCS-Y	2.793E-05	0.132	-5.658E-06	6.152E-05	2.666E-05	0.129	-5.251E-06	5.857E-05
		HC	**4.040E-5[*]**	**0.012**	**7.266E-06**	**7.353E-05**	**4.145E-5[*]**	**0.006**	**9.975E-06**	**7.293E-05**
	HC	FBTCS-Y	-1.247E-05	0.636	-3.706E-05	1.213E-05	-1.479E-05	0.363	-3.816E-05	8.574E-06
T_Postcentral (TPOSTC)	FBTCS-N	FBTCS-Y	1.192E-05	0.602	-1.101E-05	3.485E-05	1.068E-05	0.922	-1.517E-05	3.653E-05
		HC	**2.643E-5[*]**	**0.017**	**3.806E-06**	**4.905E-05**	2.075E-05	0.146	-4.747E-06	4.624E-05
	HC	FBTCS-Y	-1.451E-05	0.110	-3.130E-05	2.284E-06	-1.007E-05	0.572	-2.899E-05	8.855E-06
T_Precentral (TPREC)	FBTCS-N	FBTCS-Y	1.486E-05	0.443	-1.032E-05	4.004E-05	1.386E-05	0.576	-1.228E-05	4.001E-05
		HC	**3.019E-5[*]**	**0.013**	**5.349E-06**	**5.503E-05**	**2.818E-5[*]**	**0.028**	**2.396E-06**	**5.397E-05**
	HC	FBTCS-Y	-1.533E-05	0.132	-3.377E-05	3.108E-06	-1.432E-05	0.206	-3.346E-05	4.822E-06
T_Prefrontal (TPREF)	FBTCS-N	FBTCS-Y	1.849E-05	0.202	-6.092E-06	4.308E-05	1.947E-05	0.179	-5.651E-06	4.459E-05
		HC	**3.034E-5[*]**	**0.010**	**6.088E-06**	**5.459E-05**	**3.403E-5[*]**	**0.004**	**9.254E-06**	**5.881E-05**
	HC	FBTCS-Y	-1.185E-05	0.323	-2.985E-05	6.156E-06	-1.456E-05	0.164	-3.296E-05	3.831E-06
T_Premotor (TPREM)	FBTCS-N	FBTCS-Y	1.962E-05	0.141	-4.315E-06	4.356E-05	1.978E-05	0.135	-4.115E-06	4.367E-05
		HC	**3.026E-5[*]**	**0.008**	**6.646E-06**	**5.387E-05**	**3.426E-5[*]**	**0.002**	**1.069E-05**	**5.782E-05**
	HC	FBTCS-Y	-1.063E-05	0.411	-2.816E-05	6.893E-06	-1.448E-05	0.135	-3.198E-05	3.009E-06
Uncinate fascicle (UF)	FBTCS-N	FBTCS-Y	1.257E-05	0.685	-1.315E-05	3.830E-05	1.055E-05	1.000	-1.790E-05	3.901E-05
		HC	2.527E-05	0.051	-1.033E-07	5.064E-05	2.612E-05	0.075	-1.946E-06	5.418E-05
	HC	FBTCS-Y	-1.270E-05	0.299	-3.153E-05	6.139E-06	-1.557E-05	0.207	-3.640E-05	5.266E-06

			TW-AD							
			**LEFT**				**RIGHT**			
**Tract**	**Group 1**	**Group 2**	**Mean Difference**	***p***	**95% CI Lower**	**95% CI Upper**	**Mean Difference**	***p***	**95% CI Lower**	**95% CI Upper**
Anterior thalamic radiation (ATR)	FBTCS-N	FBTCS-Y	1.322E-05	0.932	-1.901E-05	4.545E-05	2.001E-05	0.344	-1.103E-05	5.106E-05
		HC	2.248E-05	0.254	-9.307E-06	5.427E-05	2.630E-05	0.114	-4.319E-06	5.693E-05
	HC	FBTCS-Y	-9.262E-06	0.995	-3.286E-05	1.433E-05	-6.292E-06	1.000	-2.902E-05	1.644E-05
Superior thalamic radiation (STR)	FBTCS-N	FBTCS-Y	1.475E-05	1.000	-2.419E-05	5.369E-05	-3.687E-07	1.000	-3.992E-05	3.918E-05
		HC	1.987E-05	0.609	-1.854E-05	5.828E-05	1.305E-05	1.000	-2.596E-05	5.205E-05
	HC	FBTCS-Y	-5.116E-06	1.000	-3.363E-05	2.340E-05	-1.341E-05	0.759	-4.237E-05	1.554E-05
Corticospinal (CST)	FBTCS-N	FBTCS-Y	1.729E-05	0.502	-1.348E-05	4.805E-05	1.133E-05	1.000	-1.864E-05	4.130E-05
		HC	1.870E-05	0.393	-1.165E-05	4.904E-05	1.500E-05	0.634	-1.456E-05	4.456E-05
	HC	FBTCS-Y	-1.410E-06	1.000	-2.394E-05	2.112E-05	-3.668E-06	1.000	-2.561E-05	1.827E-05
Fronto pontine (FPT)	FBTCS-N	FBTCS-Y	1.739E-05	0.439	-1.197E-05	4.675E-05	1.469E-05	0.582	-1.314E-05	4.252E-05
		HC	1.985E-05	0.283	-9.116E-06	4.881E-05	2.004E-05	0.226	-7.413E-06	4.748E-05
	HC	FBTCS-Y	-2.459E-06	1.000	-2.396E-05	1.904E-05	-5.347E-06	1.000	-2.572E-05	1.503E-05
Parieto-occipital pontine (POPT)	FBTCS-N	FBTCS-Y	2.010E-05	0.229	-7.526E-06	4.772E-05	1.924E-05	0.370	-1.134E-05	4.983E-05
		HC	1.751E-05	0.347	-9.738E-06	4.475E-05	1.992E-05	0.319	-1.025E-05	5.009E-05
	HC	FBTCS-Y	2.588E-06	1.000	-1.764E-05	2.281E-05	-6.728E-07	1.000	-2.307E-05	2.172E-05
Inferior cerebellar peduncle (ICP)	FBTCS-N	FBTCS-Y	-7.760E-06	1.000	-3.507E-05	1.955E-05	-3.925E-07	1.000	-2.915E-05	2.836E-05
		HC	5.743E-06	1.000	-2.119E-05	3.268E-05	1.429E-05	0.644	-1.408E-05	4.265E-05
	HC	FBTCS-Y	-1.350E-05	0.297	-3.350E-05	6.491E-06	-1.468E-05	0.267	-3.573E-05	6.375E-06
Superior cerebellar peduncle (SCP)	FBTCS-N	FBTCS-Y	-1.324E-05	1.000	-5.165E-05	2.518E-05	-1.043E-05	1.000	-4.622E-05	2.536E-05
		HC	1.201E-05	1.000	-2.588E-05	4.990E-05	1.242E-05	1.000	-2.288E-05	4.772E-05
	HC	FBTCS-Y	-2.525E-05	0.091	-5.338E-05	2.874E-06	-2.285E-05	0.106	-4.905E-05	3.352E-06
Inferior fronto occipital (IFO)	FBTCS-N	FBTCS-Y	2.489E-05	0.145	-5.660E-06	5.544E-05	**3.259E-5***	**0.028**	**2.794E-06**	**6.240E-05**
		HC	2.661E-05	0.099	-3.523E-06	5.675E-05	**3.529E-5***	**0.014**	**5.894E-06**	**6.468E-05**
	HC	FBTCS-Y	-1.720E-06	1.000	-2.409E-05	2.065E-05	-2.694E-06	1.000	-2.451E-05	1.913E-05
ST_ Fronto orbital (STFO)	FBTCS-N	FBTCS-Y	1.016E-05	1.000	-2.340E-05	4.373E-05	2.445E-05	0.231	-9.248E-06	5.814E-05
		HC	1.824E-05	0.528	-1.488E-05	5.135E-05	2.816E-05	0.121	-5.080E-06	6.139E-05
	HC	FBTCS-Y	-8.071E-06	1.000	-3.265E-05	1.651E-05	-3.708E-06	1.000	-2.838E-05	2.096E-05
ST_Occipital (STOCC)	FBTCS-N	FBTCS-Y	3.642E-05	0.074	-2.529E-06	7.537E-05	3.151E-05	0.086	-3.189E-06	6.620E-05
		HC	3.264E-05	0.120	-5.779E-06	7.105E-05	**4.260E-5***	**0.010**	**8.379E-06**	**7.683E-05**
	HC	FBTCS-Y	3.782E-06	1.000	-2.473E-05	3.230E-05	-1.110E-05	0.843	-3.650E-05	1.431E-05
ST_Parietal (STPAR)	FBTCS-N	FBTCS-Y	2.300E-05	0.155	-5.689E-06	5.168E-05	2.618E-05	0.102	-3.606E-06	5.596E-05
		HC	2.078E-05	0.221	-7.511E-06	4.908E-05	2.489E-05	0.121	-4.486E-06	5.427E-05
	HC	FBTCS-Y	2.213E-06	1.000	-1.879E-05	2.322E-05	1.286E-06	1.000	-2.052E-05	2.309E-05
ST_Postcentral (STPOSTC)	FBTCS-N	FBTCS-Y	1.260E-05	0.946	-1.841E-05	4.361E-05	1.384E-05	0.931	-1.988E-05	4.757E-05
		HC	1.867E-05	0.404	-1.192E-05	4.926E-05	1.933E-05	0.461	-1.393E-05	5.260E-05
	HC	FBTCS-Y	-6.076E-06	1.000	-2.878E-05	1.663E-05	-5.490E-06	1.000	-3.018E-05	1.920E-05
ST_Precentral (STPREC)	FBTCS-N	FBTCS-Y	1.550E-05	0.654	-1.550E-05	4.650E-05	1.309E-05	0.930	-1.878E-05	4.497E-05
		HC	2.288E-05	0.206	-7.695E-06	5.346E-05	2.087E-05	0.314	-1.056E-05	5.231E-05
	HC	FBTCS-Y	-7.383E-06	1.000	-3.008E-05	1.531E-05	-7.781E-06	1.000	-3.112E-05	1.555E-05
ST_Prefrontal (STPREF)	FBTCS-N	FBTCS-Y	1.616E-05	0.541	-1.350E-05	4.583E-05	1.979E-05	0.306	-9.793E-06	4.937E-05
		HC	2.212E-05	0.198	-7.136E-06	5.138E-05	2.555E-05	0.103	-3.621E-06	5.473E-05
	HC	FBTCS-Y	-5.957E-06	1.000	-2.768E-05	1.576E-05	-5.768E-06	1.000	-2.743E-05	1.589E-05
ST_Premotor (STPREM)	FBTCS-N	FBTCS-Y	1.549E-05	0.701	-1.656E-05	4.754E-05	1.415E-05	0.747	-1.611E-05	4.440E-05
		HC	2.479E-05	0.171	-6.820E-06	5.641E-05	2.191E-05	0.221	-7.933E-06	5.175E-05
	HC	FBTCS-Y	-9.307E-06	0.980	-3.277E-05	1.416E-05	-7.762E-06	1.000	-2.991E-05	1.439E-05
T_Occipital (TOCC)	FBTCS-N	FBTCS-Y	3.761E-05	0.087	-3.851E-06	7.906E-05	2.624E-05	0.236	-1.013E-05	6.261E-05
		HC	3.376E-05	0.137	-7.130E-06	7.465E-05	**4.146E-5***	**0.019**	**5.583E-06**	**7.733E-05**
	HC	FBTCS-Y	3.844E-06	1.000	-2.651E-05	3.420E-05	-1.521E-05	0.482	-4.184E-05	1.141E-05
T_Postcentral (TPOSTC)	FBTCS-N	FBTCS-Y	1.219E-05	0.999	-1.895E-05	4.334E-05	1.060E-05	1.000	-2.439E-05	4.559E-05
		HC	1.774E-05	0.468	-1.297E-05	4.846E-05	1.910E-05	0.521	-1.541E-05	5.362E-05
	HC	FBTCS-Y	-5.550E-06	1.000	-2.835E-05	1.725E-05	-8.503E-06	1.000	-3.412E-05	1.712E-05
T_Precentral (TPREC)	FBTCS-N	FBTCS-Y	1.569E-05	0.657	-1.576E-05	4.714E-05	8.954E-06	1.000	-2.316E-05	4.107E-05
		HC	2.209E-05	0.247	-8.930E-06	5.311E-05	1.960E-05	0.388	-1.207E-05	5.128E-05
	HC	FBTCS-Y	-6.401E-06	1.000	-2.943E-05	1.663E-05	-1.065E-05	0.791	-3.416E-05	1.286E-05
T_Prefrontal (TPREF)	FBTCS-N	FBTCS-Y	1.635E-05	0.553	-1.394E-05	4.663E-05	1.754E-05	0.440	-1.211E-05	4.720E-05
		HC	2.322E-05	0.177	-6.656E-06	5.309E-05	2.562E-05	0.103	-3.626E-06	5.488E-05
	HC	FBTCS-Y	-6.870E-06	1.000	-2.904E-05	1.530E-05	-8.080E-06	1.000	-2.979E-05	1.363E-05
T_Premotor (TPREM)	FBTCS-N	FBTCS-Y	1.357E-05	0.926	-1.937E-05	4.651E-05	1.022E-05	1.000	-1.939E-05	3.982E-05
		HC	2.446E-05	0.201	-8.032E-06	5.696E-05	2.172E-05	0.211	-7.480E-06	5.093E-05
	HC	FBTCS-Y	-1.089E-05	0.796	-3.501E-05	1.323E-05	-1.150E-05	0.575	-3.318E-05	1.017E-05
Uncinate fascicle (UF)	FBTCS-N	FBTCS-Y	1.911E-05	0.514	-1.522E-05	5.344E-05	3.543E-05	0.059	-1.026E-06	7.188E-05
		HC	2.269E-05	0.304	-1.117E-05	5.655E-05	3.042E-05	0.122	-5.539E-06	6.638E-05
	HC	FBTCS-Y	-3.578E-06	1.000	-2.871E-05	2.155E-05	5.010E-06	1.000	-2.168E-05	3.170E-05

Key: HC: control group; Mean difference: the difference between group 1 and group 2, based on the estimated marginal means of the univariate ANCOVA (Bonferroni correction selected). Positive mean difference values indicate group 1 had a higher average TW-TM than group 2, negative values indicate group 2 had a higher average TW-TM than group 1. "*p*" represents the significance of the mean difference.* a significant difference was observed in the estimated marginal means univariate ANCOVA.
